# Preliminary Anti-Melanoma Activity of a Chlorogenic Acid-Based PROTAC Targeting MDM4, a Candidate Protein Identified by Proteomics

**DOI:** 10.3390/foods15061082

**Published:** 2026-03-19

**Authors:** Zhiting Mei, Jiali Sun, Pengfei Zhao, Yiming Luo, Jine Niu, Danfei Huang

**Affiliations:** 1 State Key Laboratory of Food Science and Resources, China-Canada Joint Lab of Food Science and Technology (Nanchang), Key Laboratory of Bioactive Polysaccharides of Jiangxi Province, Nanchang University, 235 Nanjing East Road, Nanchang 330047, China; 18679466156@163.com (Z.M.); 15070878991@163.com (J.S.); 18339472400@163.com (P.Z.); luo18779602361@163.com (Y.L.); 18366061067@163.com (J.N.); 2 International Institute of Food Innovation Co., Ltd., Nanchang 330200, China

**Keywords:** natural polyphenols, proteolysis targeting chimera, quantitative proteomics, antitumor activities, apoptosis

## Abstract

Chlorogenic acid (CGA), which is ubiquitous in diverse botanical sources, demonstrates considerable anticancer potential through modulation of multiple targets or signaling pathways, thereby posing substantial challenges for mechanistic elucidation and target identification. Based on the proteolysis targeting chimera (PROTAC) technology’s ability to induce targeted protein degradation via ubiquitin-proteasome pathway recruitment, we synthesized a panel of CGA-PROTACs. These compounds incorporated the natural product CGA as the target-binding ligand, conjugated to pomalidomide (an E3 ligase-recruiting moiety) via various synthetic linkers. The findings indicated that compound **A7**, linked with an alkane linker, exhibited a notable anti-proliferative effect on 4T1 and A375 cells in vitro. The IC_50_ value of **A7** on A375 cells reached 69.70 μM, which is 2.2 times better than the effect of the precursor compound CGA (IC_50_ = 148.80 μM). Mouse double minute 4 (MDM4) was confirmed as a potential target of compound **A7** through a combination of proteomics, Western blot analysis and molecular dynamics simulation. CGA-PROTAC **A7** treatment led to a dose-dependent reduction in MDM4 protein levels while significantly upregulating p53 and p21 protein expression, and thus inhibited proliferation, induced G2/M phase cell cycle arrest, and markedly enhanced apoptosis in melanoma A375 cells. This study successfully applied an effective strategy for target identification and medication discovery of natural compounds. In addition, CGA-PROTAC **A7** was synthesized in one step with an overall yield of 45.96%, providing a feasible route for synthesis and establishing a basis for the combination of natural product polyphenols with PROTAC technology.

## 1. Introduction

Melanoma, which arises from pigment-producing melanocytes, is the most aggressive cutaneous malignancy. According to the Global Cancer Observatory, the incidence and deaths from melanoma worldwide in 2022 were 331,000 and 58,000, respectively [1]. Patients with localized melanoma have a 99% five-year survival rate, while this rate decreases to 38% for cases with distant metastases [2]. Surgical removal represents the standard therapeutic approach for melanoma, although it is not curative due to the high risk of metastasis. Targeted therapy and immunotherapy offer higher survival rates but have problems with resistance, non-response, and frequent tumor relapse [3]. Chemotherapy remains an alternative treatment option, despite its potential for serious side effects. Therefore, additional research is necessary to develop innovative therapeutic agents with optimized treatment benefits and reduced side effects. Indeed, natural products have gained significant attention recently due to their specificity and safety in melanoma therapy [4]. However, structural modifications of natural products are sometimes necessary before they can be transformed into therapeutic agents to tackle possibly unfavorable physicochemical properties and restricted biological activity [5]. More importantly, it is challenging to elucidate binding sites of most natural products.

Chlorogenic acid (CGA), a natural polyphenol belonging to phenylpropanoids with polyhydroxyphenol groups, is found in numerous plants and exhibits significant anticancer potential [6]. Kimsa-Dudek et al. showed that CGA reduces the growth and intensifies the oxidative stress in melanoma C32 cells by inhibiting the expression and activity of antioxidant enzymes such as SOD and GSH-Px [7]. Moreover, Li et al. showed that combining a PD-1 antibody with sialic acid-functionalized liposomes (CA-SAL) enhanced T-cell immunity. The treatment elevated CD8^+^ T cells and M1 macrophage levels, which effectively controlled the proliferation and progression of B16F10 melanoma cells [8]. Recently, Li et al. reported that CGA augments T cell-based antitumor immunity via down-regulation of PD-L1 through the IFN-γ/JAK/pSTAT1/IRF1 pathway [9]. While CGA has been reported to exert phenotypic effects (oxidative stress, immune modulation) in melanoma cells, its anti-melanoma activity is weak and its molecular target remains unidentified. Moreover, the lack of detailed research to identify its significant target proteins has limited its development as a potential therapeutic agent.

The PROTAC technology leverages the cellular degradation machinery to induce ubiquitination and degrade specific proteins of interest (POIs), offering a novel approach for targeted protein modulation. Typically, PROTACs are trimeric complexes composed of a ligand that binds the POI, a ligand for an E3 ubiquitin ligase, and a chemical linker that connects these two ligands [10,11]. The emergence of this technique also provides a reliable method for identifying the target proteins of small molecular compounds [12,13,14]. Degrading an oncogenic protein in cancer cells is theoretically more effective than inhibiting it [15]. PROTACs based on natural products can effectively enhance the activity of natural products and be used to identify drug targets [16].

Therefore, we envision that converting CGA into a PROTAC would enable simultaneous target identification (proteomics) and enhanced anti-melanoma activity (targeted protein degradation). Based on structure–activity relationship (SAR) investigations of chlorogenic acid analogs, modification at the carboxylic acid moiety of quinic acid has been shown to preserve its inherent bioactivity, and chemical probes exploiting this site have been successfully employed to capture the protein targets of chlorogenic acid [17]. Leveraging this insight, we designed and synthesized ten CGA-based PROTACs functionalized at this position and assessed their antiproliferative effects in tumor cells. Through an integrated approach combining quantitative proteomics, immunoblotting, and computational modeling, MDM4 was preliminarily identified as a potential target protein. This work offers a useful strategy for target identification and medication discovery from natural products.

## 2. Materials and Methods

### 2.1. Materials

Chlorogenic acid (CAS 327-97-9, purity ≥ 98%) used in this study was purchased from Yuanye Biotechnology Co., Ltd. (Shanghai, China). The intermediate compounds **C1**–**C10** (purity ≥ 90%) were obtained from Zhengzhou Alfachem Chemical Co., Ltd. (Zhengzhou, China). Unless otherwise indicated, all reagents were obtained from commercial sources and employed without additional purification. The HepG2, 4T1, A375 and HEK293 cell lines used were cryopreserved in our laboratory. All cell lines were grown in DMEM (Solarbio, Beijing, China) supplemented with 10% FBS (Vivacell, Shanghai, China) and 1% penicillin-streptomycin solution (Solarbio, Beijing, China). All cells were incubated at 37 °C in a 5% CO_2_ incubator.

### 2.2. Producing of ***A1***–***A10*** Compounds

The general synthesis process and characterization of the compounds were performed according to the protocols of classical synthesis with some modifications [18]. Briefly, intermediates **C1**–**C10** were separately dissolved in 1 mL of DMF/tetrahydrofuran/acetonitrile (1:1:1, *v*/*v*/*v*), DIPEA (0.1 eq) was then added, mixed thoroughly, and subjected to ultrasonication to ensure complete dissociation. Chlorogenic acid (0.1 eq) was dissolved in 1 mL of tetrahydrofuran/acetonitrile mixture (1:1, *v*/*v*). Subsequently, EDCI (0.12 eq), HOBT (0.12 eq), and carboxymethyl cellulose sodium (CMC) (0.1 eq) were added. The mixture was maintained under stirring in an ice–salt bath for 30 min. The previously prepared **C1**–**C10** solutions were slowly added separately, followed by DIPEA (0.2 eq), and the reaction was maintained under nitrogen for 10 h. The progress of all reactions was monitored by thin-layer chromatography (TLC) analysis using GF254 silica gel plates (Qingdao Ocean Chemical Co., Ltd., Qingdao, China). After reactions were complete, reaction mixtures underwent extraction with ethyl acetate, followed by washing with saturated brine (2–3 times). Drying of the combined organic phases with anhydrous Na_2_SO_4_, followed by filtration and concentration under reduced pressure using a rotary evaporator, was performed (EYELASB-1000, Tokyo Rikakikai Co., Ltd., Tokyo, Japan). The residues underwent purification via silica gel column chromatography with a gradient of dichloromethane and methanol (20:1, *v*/*v*) to yield yellow oily substances, which were further purified by preparative TLC to obtain pale yellow powder compounds **A1**–**A10**. The purity of the compounds was analyzed using a Shimadzu Nexera UHPLC LC-30A system (Shimadzu Corporation, Kyoto, Japan) coupled with a Sciex 5600+ QTOF (Sciex, Framingham, MA, USA) mass spectrometer (UHPLC-QTOF-MS). ^1^H NMR and ^1^C NMR spectra were recorded on a Bruker AVANCE NEO 600 MHz NMR spectrometer (Bruker Switzerland AG, Fallanden, Switzerland), with chemical shifts reported in ppm. Detailed NMR and LCMS spectra can be found in Supplementary Materials.

### 2.3. In Vitro Cytotoxicity Assay

The CCK-8 assay was employed to assess the anti-proliferative effects of the compounds in vitro. HepG2, 4T1, A375 and HEK293 cells were seeded at a density of 5 × 10^3^ cells per well in 96-well plates and cultured overnight. Subsequently, 100 μL of medium in each well was substituted with 100 μL of medium containing 100 μM PROTACs or the positive control compound arecoline hydrobromide (AH) for an additional 48 h, whereas the negative control group received a medium supplemented with an equivalent volume of DMSO. Then, 10 μL of CCK-8 solution was added. After a designated period, the samples were further incubated. Absorbance at 450 nm was determined using a Varioskan Flash microplate reader (Thermo Scientific, Waltham, MA, USA).

### 2.4. DIA-Mass Spectrometry for Proteomics

A375 cells were seeded in 100-mm dishes at a density of 2 × 10^5^ cells/mL and cultured overnight. Cells were exposed to compound **A7** (0 or 100 μM) for 48 h; three biological replicates per group were collected and analyzed using the DIA-based quantitative proteomic analysis (Shanghai Biotree Biotech Co., Ltd., Shanghai, China).

### 2.5. Clonogenic Assays

A375 cells were plated in a 6-well plate at a density of 2,000 cells per well and cultured overnight. Then compound **A7** at final concentrations of 0, 50, 75 and 100 µM was added and co-incubated for an additional 14 days, during which, the medium in each well was changed every three days and cellular morphology and colony formation were assessed. When a single clone colony with more than 50 cells was formed, the culture medium was aspirated, and the cells were washed twice with PBS, fixed in 4% paraformaldehyde (15 min), and stained with 0.1% crystal violet for 10 min. Finally, images were captured and colonies containing more than 50 individual cells were subsequently quantified employing ImageJ software (version 1.54k, National Institutes of Health, Bethesda, MD, USA).

### 2.6. Calcein-AM/PI Cell Double Staining Assay

A375 cells were plated in 24-well plates at a density of 5 × 10^4^ cells/mL overnight followed by treatment with compound **A7** at concentrations of 0, 50, 75 and 100 µM at the end of 48 h. After the medium was aspirated, the cells were washed with PBS and then incubated with the Calcein-AM/PI Live/Dead Assay Kit (Beyotime, Shanghai, China) for 30 min at 37 °C. Finally, fluorescence images were acquired using an inverted fluorescence microscope (Leica Microsystems, Heidelberg, Germany).

### 2.7. Scratch Wound Healing Assay

A375 cells (3 × 10^5^ cells/mL) were seeded into 12-well plates and incubated overnight to form a confluent monolayer. The culture medium was then changed to DMEM containing 5% FBS for a starving period of 48 h. A scratch wound was made and the cells were treated with compound **A7** at final concentrations of 0, 50, 75 and 100 µM after washing 3 times with PBS to remove debris and non-adherent cells. Wound area images were captured using phase contrast microscopy at 0, 24 and 48 h after treatment and quantitatively analyzed with ImageJ software (version 1.54k, National Institutes of Health, Bethesda, MD, USA).

### 2.8. Cell Cycle Analysis

Cells were plated in 6-well plates at a density of 2 × 10^5^ cells/mL and incubated overnight to adhere. After treating with varying doses of compound **A7** (0–100 μM) for 48 h, cells were washed with pre-cooled PBS and then fixed with 70% pre-cooled ethanol overnight at 4 °C. Subsequently, cells were stained with propidium iodide (PI) solution according to the manufacturer’s instructions (Beyotime, Shanghai, China), followed by analysis using DXFLEX Plus flow cytometry (Beckman Coulter, Brea, CA, USA).

### 2.9. Cell Apoptosis Analysis

Cells (2 × 10^5^ cells/mL) were seeded in 6-well plates and administered specified concentrations (0, 50, 75, and 100 µM) of compound **A7** for 48 h. Following incubation, cellular apoptosis was determined with the Annexin V-FITC/PI Apoptosis Detection Kit (Beyotime, Shanghai, China), with subsequent analysis conducted using a DxFLEX Plus flow cytometer (Beckman Coulter, Brea, CA, USA).

### 2.10. Western Blotting

After incubation with compound **A7** at specified concentrations (0–100 µM) for 48 h, cells were collected, washed with cold PBS, and lysed on ice for 30 min using lysis buffer supplemented with protease inhibitor, PMSF and phosphatase inhibitor. Following quantifying protein concentrations via the Bradford assay, proteins were resolved by 10% SDS-PAGE and transferred to PVDF membranes. Following a 15-min blocking step at room temperature, the membranes were incubated overnight at 4 °C with primary antibodies against MDM4 rabbit antibody (28747-1-AP, Proteintech, Wuhan, China)) and MDM2 rabbit antibody (EPR22256-98, Abcam, Cambridge, UK), p53 rabbit antibody (TA0879, Abmart, Shanghai, China), rabbit monoclonal antibody to p21 (Abmart, T55543), Bax rabbit antibody (Abmart, T40051), Bcl-2 rabbit mAb (Abmart, T40056), and β-actin mouse antibody (TA-09, ZSGB-BIO, Beijing, China). Following thorough washes with TBST, membranes were incubated with HRP-conjugated anti-rabbit secondary antibody (ZSGB-BIO, ZB-2301) or anti-mouse secondary antibody (ZSGB-BIO, ZB-5305). The hybridization bands were ultimately detected using a Gel Doc XR system (Bio-Rad Laboratories, Hercules, CA, USA).

### 2.11. Molecular Docking

The crystal structure of MDM4 (PDB code: 3FEA) was downloaded from the Protein Data Bank database of the NCBI, and the structure of CRBN (PDB code: 5FQD) was retrieved from the same source. For the preparation of ligand structures, compound **A7**’s structure was generated using ChemDraw software (version 22.0) and subsequently exported into PDB format via Open Babel (version 2.4). For binary complex docking, the MDM4 and **A7** structures were processed using AutoDock Vina (version 1.2), with a grid box centered on the p53-binding pocket of MDM4 based on the co-crystallized peptide in the 3FEA structure, and docking parameters were set to default values. To model the ternary complex, the docked MDM4–**A7** binary complex and the CRBN structure were submitted to the HDOCK (version 1.1) protein–protein docking server. Since no prior information regarding the binding interface was available, an ab initio global docking approach was employed to sample the six-dimensional (three translational and three rotational) conformational space of the complex. The top-scoring ternary complex model was selected based on the HDOCK docking score. Subsequently, the Analyze module of AutoDock Vina was employed to conduct interaction force analysis on both binary and ternary complexes, and molecular visualization was performed using PyMOL software (version 2.5) to generate structural representations.

### 2.12. Molecular Dynamics Simulations

Molecular dynamics simulations were subsequently conducted using the GROMACS software package (version 2022) to gain deeper insights into the binary structure (MDM4/**A7**) and the stability of ternary (MDM4/**A7**/CRBN) complexes under physiological conditions. Molecular mechanics parameters were generated using the GROMACS tool pdb2gmx, while the molecular parameters of ligands were provided by AutoFF, an automated web-based tool. The following metrics were calculated using GROMACS utilities to evaluate structural changes and solvent effects for system stability analysis: root-mean-square deviation (RMSD) via gmx-rmsd. Structural stability and flexibility were evaluated by calculating the root-mean-square fluctuation (RMSF), number of hydrogen bonds (HBonds), radius of gyration (Rg), and solvent-accessible surface area (SASA) using the gmx rmsf, gmx hbond, gmx gyrate, and gmx sasa tools, respectively.

### 2.13. Statistical Analysis

All experiments were performed independently in triplicate. Data were processed using SPSS Statistics 27.0 and expressed as mean ± standard deviation (SD). Graphical illustrations were generated with GraphPad Prism 10.1. Statistical comparisons between multiple groups were conducted using one-way analysis of variance (ANOVA), with *p* < 0.05 considered statistically significant. Prior to statistical analysis, the normality and homogeneity of variance of the data were verified, and Tukey HSD or Dunnett’s T3 tests were used based on the homogeneity of variance results. Statistical significance relative to the negative control group is indicated as follows: * *p* < 0.05, ** *p* < 0.01, *** *p* < 0.001, ns: not significant.

## 3. Results

### 3.1. Design and Synthesis of CGA-Based PROTACs

**Figure 1:**
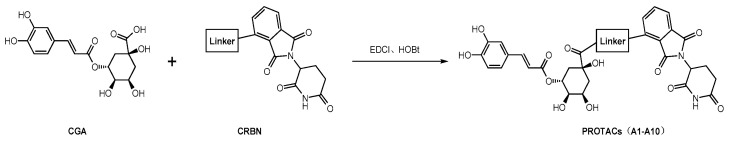
General synthetic route of CGA-based PROTACs.

Evidence from CGA analog SAR suggests that the quinic acid carboxyl group can be modified while retaining full biological function [19,20]. Consequently, the carboxylic acid group was selected as a suitable modification site to facilitate the attachment of the linker segment. Pomalidomide was chosen as the E3 ligase cereblon (CRBN) ligand, which exhibits inherent advantages including specific and robust binding affinity for its target E3 ligases, acceptable physicochemical properties, and well-characterized structural information regarding its binding modes [21]. Given the significant correlation between the length and type of the linker and PROTACs’ efficacy [22], a series of these compounds using alkyl chains, polyethylene glycol (PEG) chains, or heterocycle chains with varying linker lengths was meticulously designed and subsequently synthesized (Figure 1). The amide bond formations between the CGA and pomalidomide linker components were executed employing EDCI/HOBt as the coupling agents, resulting in the production of compounds **A1**–**A10**. These compounds were obtained with yields ranging from 8.94% to 45.96% and exhibited a purity of 90.71% to 95.34% subsequent to chromatographic purification (Table 1). The structures of CGA derivatives were characterized by ^1^H NMR, ^1^C NMR and LCMS.

### 3.2. Effects of CGA-Based PROTACs on the Proliferation of A375, 4T1 and HepG2 Cells

**Figure 2:**
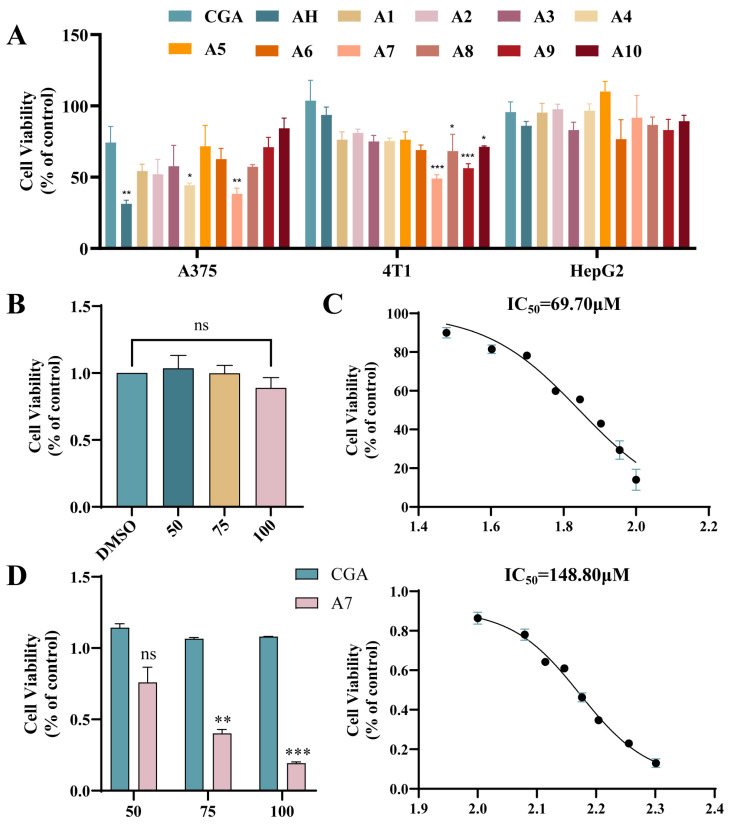
(**A**) Cell proliferation assay in three cell lines (n = 3). * *p* < 0.05, ** *p* < 0.01, *** *p* < 0.001, ns: not significant, compared with the CGA group of each cell line; (**B**) Cell viability levels of HEK293 cells treated with different concentrations of **A7**; (**C**) IC_50_ values of CGA and **A7** compounds (n = 3); (**D**) Comparison of activity of CGA and **A7** on A375 cells at the same concentration.

As presented in Figure 2, all the CGA-PROTACs demonstrated moderate to outstanding antitumor activity against 4T1 and A375 cells, yet displayed no cytotoxicity towards HepG2 cells, which express low levels of MDM4 [23]. Specifically, for compounds tethered via alkyl or PEG unit linkers, antiproliferative activity increased with linker extension. Among them, **A4** with 4 PEG and **A7** with 6 alkyl linkers had significant anti proliferative activity. On the other hand, compound **A7** with a six-carbon chain exhibited the best antitumor activity against A375 cells (IC_50_ = 69.70 μM), which was approximately 2–3 times more potent than the parent CGA’s (IC_50_ = 148.80 μM) anti-A375 activity. Furthermore, **A7** exhibited no significant cytotoxicity toward HEK293 cells at any of the tested concentrations, indicating that its antiproliferative effect is attributable to specific PROTAC-mediated activity rather than non-specific toxicity at high doses (Figure 2B). Therefore, modification techniques based on PROTAC can enhance the antiproliferative activity of weak natural product ligands.

### 3.3. CGA-PROTAC ***A7*** Induced a Reduction in MDM4 Protein Levels via the UPS

**Figure 3:**
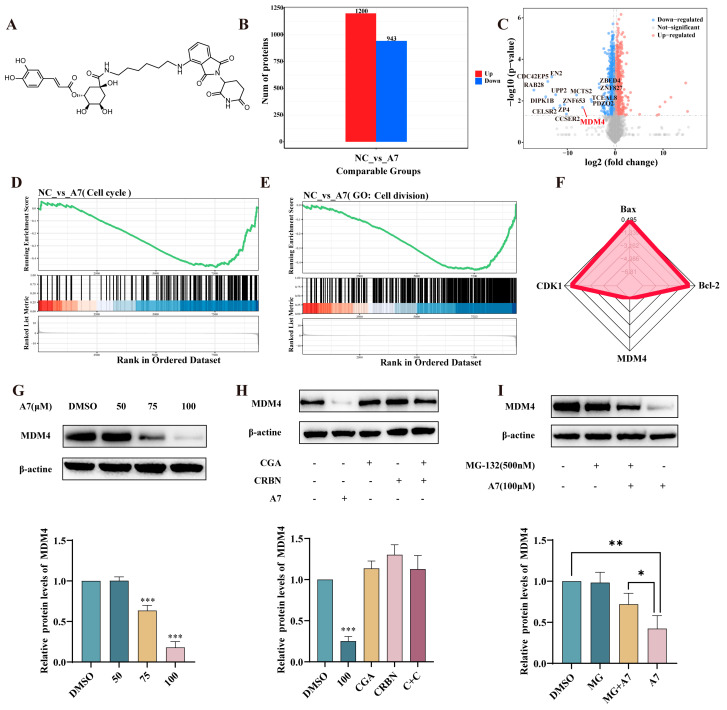
MDM4 is a potential target of CGA-PROTAC **A7**. (**A**) CGA-PROTAC **A7** structure; (**B**) Quantitative proteomic analysis revealing up-regulated (red) and down-regulated (blue) proteins in A375 cells after 48-h exposure to 100 μM **A7** relative to untreated controls; (**C**) Volcano Plot highlighting differentially expressed proteins between CGA-PROTAC **A7** and negative control; (**D**,**E**) GSEA results for cell cycle and cell division; (**F**) Radar chart deviation analysis of MDM4, Bax, Bcl-2 and CDK2; (**G**) Dose-responsive reduction in MDM4 induced by CGA-PROTAC **A7**; (**H**) MDM4 levels normalized to β-actin in A375 cells following 48-h treatment with either CGA-PROTAC **A7** (100 μM), CGA (100 μM), CRBN ligand (100 μM), or a mixture of CGA plus CRBN ligand (n = 3); (**I**) MDM4 levels in A375 cells after a 2-h MG132 (500 nM) pretreatment and a subsequent 48-h exposure to DMSO or 100 μM CGA-PROTAC **A7** (n = 5). * *p* < 0.05, ** *p* < 0.01, *** *p* < 0.001 compared with the negative control group (DMSO).

Target proteins are selectively degraded by PROTACs, and reduced protein expression levels can be accurately measured using advanced proteomic analysis techniques, such as mass spectrometry-based quantitative proteomics [24]. As a result, this method makes it possible to identify potential targets for natural products. As shown in Figure 3B, 1200 upregulated and 943 downregulated proteins were identified. Among these, multiple proteins exhibited significant downregulation in compound **A7**-treated cells (log fold change < −3, *p* < 0.05). After consulting the protein-related information on the UniProt website, we found that only MDM4 protein is closely related to cancer, with its protein level downregulated by nearly 7 times and overexpressed in various cancer cells such as A375 cells. Therefore, we preliminarily speculated that MDM4 is the potential target protein. Pathway enrichment analysis revealed significant downregulation of pathways associated with cell cycle regulation, cell division, and DNA replication, indicating significantly suppressed cell proliferation activity and cell cycle arrest (Figure 3D,E). The pro-apoptotic shift in the Bax/Bcl-2 ratio observed upon **A7** treatment is consistent with the induction of apoptosis. Western blotting analysis was employed to further assess the effect of CGA-PROTAC **A7** on MDM4 protein levels in A375 cells. As illustrated in Figure 3G, CGA-PROTAC **A7** treatment resulted in a dose-dependent reduction in MDM4 protein expression. Neither CGA, the CRBN ligand pomalidomide, nor their mixture decreased MDM4 protein level, whereas CGA-PROTAC **A7** at an equivalent concentration significantly reduced the expression level of MDM4 in A375 cells (Figure 3H). These findings suggested that MDM4 reduction could be selectively induced by CGA-PROTAC **A7**. Moreover, pretreatment of A375 cells with the proteasome inhibitor MG132 was conducted to ascertain whether CGA-PROTAC **A7** facilitated MDM4 protein reduction through the ubiquitin-proteasome system (UPS). As depicted in Figure 3I, MG132 pretreatment alleviated the reduction in MDM4, indicating that the reduction in MDM4 induced by CGA-PROTAC **A7** was dependent upon the UPS.

### 3.4. Prediction of CGA-PROTAC ***A7*** Binding Mode to MDM4 Protein

**Figure 4:**
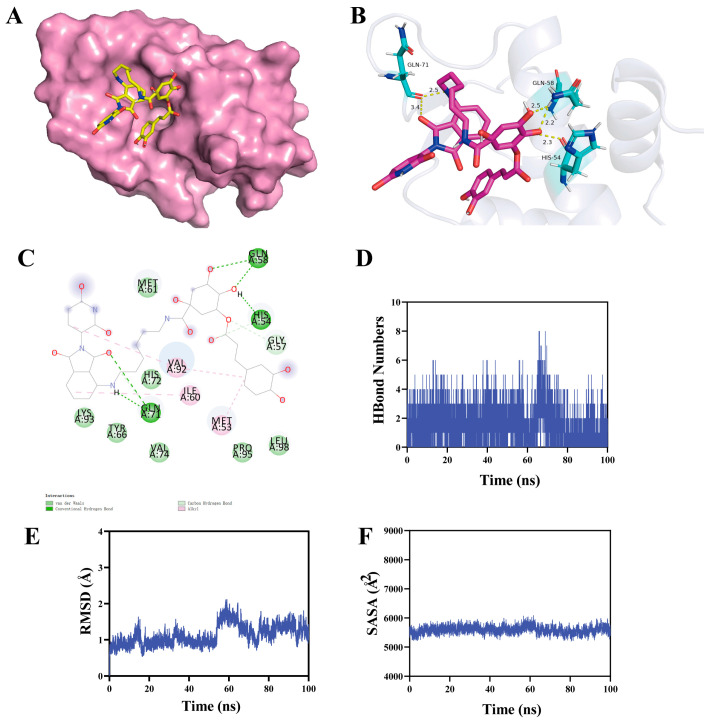
Binding mode prediction and dynamic behavior analysis of the CGA-PROTAC **A7**–MDM4 complex. (**A**) The docking pose of CGA-PROTAC **A7** in MDM4 crystal structure (PDB: 3FEA), with MDM4 shown in pink and **A7** depicted as yellow sticks; (**B**) CGA-PROTAC **A7** molecular docking modeling in the active site of receptor MDM4. The ligand molecules (red) and key residues (light green) are represented by sticks and the proteins (gray) are represented by cartoon structures. The yellow dotted line indicates a hydrogen bond; (**C**) The interaction force between the ligand and the receptor MDM4; Hydrogen bond (**D**), RMSD (**E**), and SASA (**F**) plots of the time-dependent protein-ligand interaction between CGA-PROTAC **A7** and the binding pocket of the MDM4 protein.

Molecular docking and molecular dynamics simulations were conducted to predict the binding ability between CGA-PROTAC **A7** and MDM4 protein. Docking calculations demonstrated that CGA-PROTAC **A7** forms five hydrogen bonds with specific residues (Gln-71, Gln-58, His-54) within the receptor MDM4 protein (Figure 4B). The binding energy value was −7.0 kcal/mol. In addition, molecular docking analysis revealed that compound **A7** binds within the putative active site of MDM4, forming interactions with key residues (Met-53, Leu-98, His-54, Gln-58, Gln-71, Val-74, and Tyr-66) that constitute the p53-binding pocket (Figure 4C). This binding mode is consistent with the interaction patterns reported for known MDM4 inhibitors and the native p53 peptide [25], suggesting that **A7** engages the functional ligand-binding cavity of MDM4. Molecular dynamics simulations demonstrated that the CGA-PROTAC **A7**-MDM4 protein complex system exhibited transverse fluctuations during its trajectory, achieving equilibrium after 60 ns with RMSD approximating 2 Å. This observation indicates notable structural stability of the complex. Remarkably, the CGA-PROTAC **A7**-MDM4 protein complex system exhibited no significant alterations in SASA, suggesting minimal structural perturbation induced by ligand binding. Furthermore, the complex demonstrated negligible expansion or contraction throughout its dynamic motion.

### 3.5. Computational Modeling of the MDM4–***A7***–CRBN Ternary Complex

**Figure 5:**
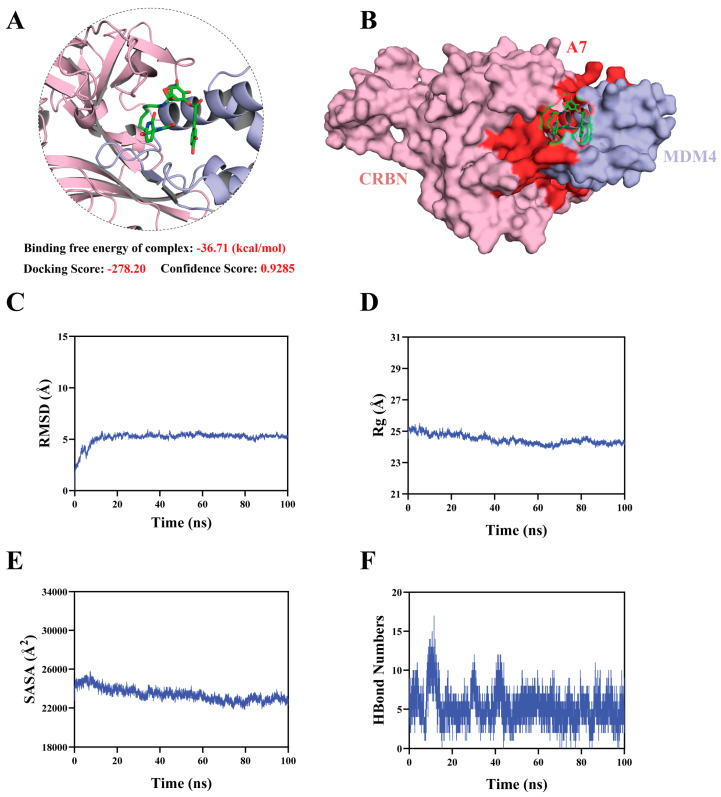
Structural modeling and conformational dynamics of the MDM4–**A7**–CRBN ternary system. (**A**) The docking result of MDM4-**A7** in CRBN crystal structure (PDB: 5FQD); (**B**) Overall structure of the ternary complex, with MDM4 shown in blue, CRBN in pink, and **A7** as green sticks; RMSD (**C**), Rg (**D**), SASA (**E**), and Hydrogen bond (**F**) plots of the time-dependent profiles of MDM4-**A7**-CRBN ternary complex.

To assess whether CGA-PROTAC **A7** can simultaneously engage MDM4 and CRBN, the ternary complex was modeled via HDOCK protein–protein docking using the pre-docked MDM4–**A7** binary complex and the CRBN structure (PDB: 5FQD), followed by 100 ns MD simulations. Docking yielded a favorable score of −278.20 and a binding free energy of −36.71 kcal/mol (confidence score: 0.9285), indicating a potentially stable ternary assembly. MD simulations showed that the ternary complex reached equilibrium within 10 ns and maintained RMSD below 5.3 Å throughout, confirming structural integrity without dissociation under the simulation conditions. Compared to the binary complex (RMSD < 2 Å), the ternary system exhibited slightly higher but stable fluctuations, consistent with its larger size and increased flexibility. Rg and SASA (Figure 5D,E) exhibited moderate fluctuations throughout the simulation, indicating that the ternary complex may undergo conformational adjustments upon ligand binding. Hydrogen bond analysis revealed persistent yet dynamic interactions (0–17 bonds, averaging 1–5), reflecting potential breathing motion of the ternary interface—a feature that could be conducive to PROTAC catalytic cycling. In contrast to the more rigid binary complex, the ternary complex displayed dynamic stability in silico, which aligned with its proposed functional role in ubiquitin transfer and E3 release. Collectively, these computational results provide structural hypotheses supporting that **A7** may assemble MDM4 and CRBN into a stable ternary complex, although further experimental validation is required to confirm this binding mode.

### 3.6. CGA-PROTAC ***A7*** Inhibited A375 Cells Proliferation and Metastasis

**Figure 6:**
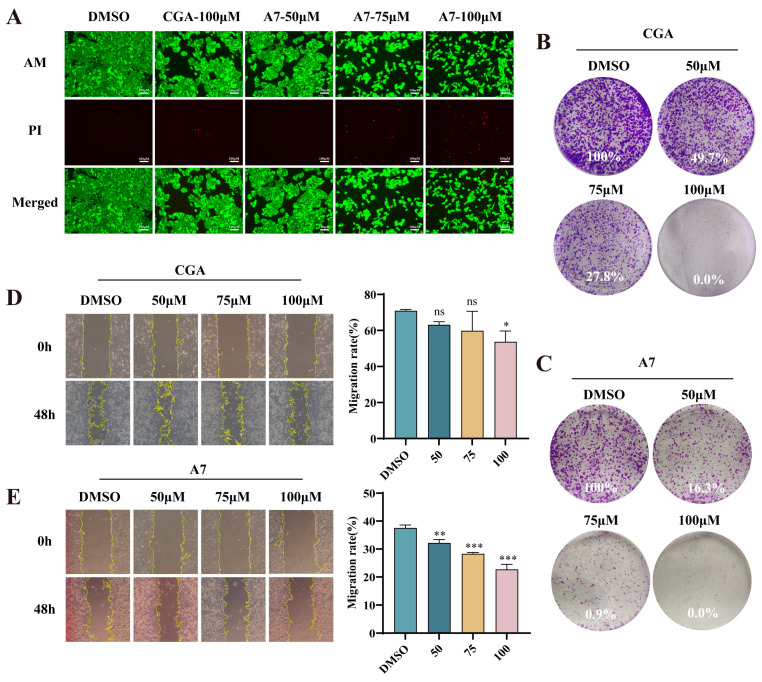
CGA-PROTAC **A7** reduces melanoma A375 cells proliferation and migration. (**A**) Representative fluorescence images of Calcein-AM and PI co-stained A375 cells treated with DMSO, CGA or CGA-PROTAC **A7** with various concentrations for 48 h; (**B**,**C**) Colony formation assays assessing clonogenicity of A375 cells after treatment with CGA or CGA-PROTAC **A7**, respectively; (**D**,**E**) Representative images and statistics of wound-healing assay for A375 cells after treatment with CGA or CGA-PROTAC **A7** with various concentrations for 48 h (n = 3). * *p* < 0.05, ** *p* < 0.01, *** *p* < 0.001, ns: no significance compared with the negative control (DMSO).

In view of research suggesting that the suppression of MDM4 expression hinders the proliferation and metastasis of tumor cells [26], the influence of CGA-PROTAC **A7** on the tumor features of A375 cells was investigated. The results of the calcein-AM/PI cell double staining assays showed that the population of live cells decreased significantly with increasing concentrations of CGA-PROTAC **A7** compared to the negative control group, while red fluorescence within the cells increased in a concentration-dependent manner, indicating the presence of dead or late apoptotic cells (Figure 6A). To determine if CGA-PROTAC **A7** can undermine the colony-forming ability of A375 cells, its activity was evaluated by clonogenic assays. At 50 μM CGA-PROTAC **A7**, colony formation was slashed to just 16.3%, whereas the clone formation of CGA at the same concentration reached 49.7%, indicating significantly increased anti-proliferative ability of CGA-PROTAC **A7** on A375 cells (Figure 6B,C). Following scratch creation, the migratory capacity of cells across the scratch was monitored at 24 h and 48 h after treatment with CGA-PROTAC **A7**. As depicted in Figure 6D,E, A375 cells showed robust migration into the scratch wound area after 48 h of incubation, with the negative control group achieving a migration rate of 38%. Nevertheless, migratory capacity exhibited concentration-dependent suppression upon CGA-PROTAC **A7** treatment, declining to merely 22% after a 48-h exposure to 100 μM CGA-PROTAC **A7**. This observation demonstrated the efficacy of CGA-PROTAC **A7** in inhibiting cancer cell motility.

### 3.7. CGA-PROTAC ***A7*** Induced Cell Apoptosis and Cycle Arrest at G2/M Phase

**Figure 7:**
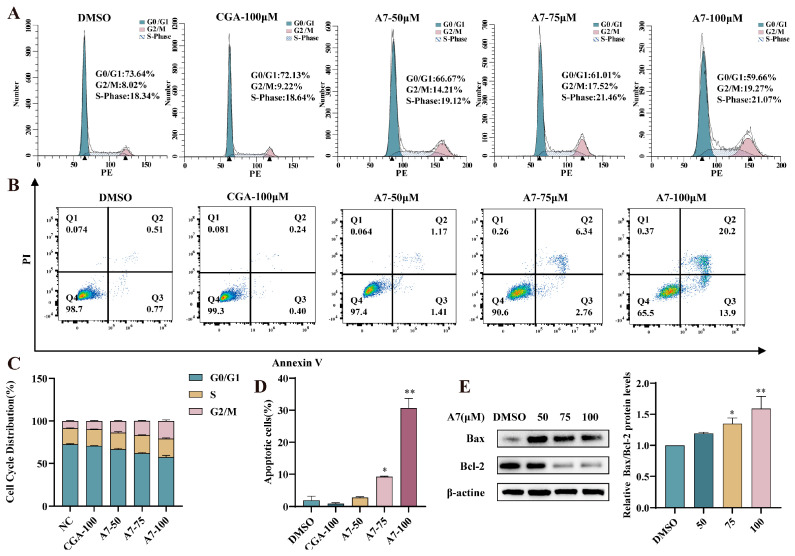
(**A**,**C**) Cell cycle analysis of A375 cells treated with CGA (100 μM) or CGA-PROTAC **A7** at different concentrations for 48 h (n = 3); (**B**,**D**) Evaluation of apoptosis rates of A375 cells incubated with CGA (100 μM) or CGA-PROTAC **A7** at different concentrations for 48 h by Annexin V/PI flow cytometry (n = 3); (**E**) Protein expression of Bax and Bcl-2 was assessed by immunoblotting, with signals normalized to β-actin (n = 3). * *p* < 0.05, ** *p* < 0.01 compared with the negative control group (DMSO).

A large body of research has established that triggering cancer cell death and disrupting the cell cycle are two fundamental ways to control tumor growth [27,28]. Proteomics has confirmed that A375 cells treated with CGA-PROTAC **A7** crank up Bax and shut down Bcl-2, two key switches that flip cells into apoptosis [29]. As depicted in Figure 7B,D, after incubation with CGA-PROTAC **A7** (50, 75 and 100 μM) for 48 h, the quantity of apoptotic cells was notably elevated to 2.5%, 9.1% and 34.1%. For cells treated with CGA alone, the apoptotic rate languished at 0.6%. The marked elevation in the Bax/Bcl-2 ratio relative to the control group demonstrates that CGA-PROTAC **A7** triggers apoptosis in A375 cells (Figure 7E). Treatment with CGA-PROTAC **A7** induced a dose-dependent accumulation of A375 cells in the G2/M phase. Specifically, the proportion of cells arrested at this checkpoint rose from 10% in the control group to 30% following a 48-h exposure to 100 μM **A7**, suggesting that significant G2/M arrest was induced. These findings collectively indicated that compound **A7** induces G2/M phase arrest in A375 cells, leading to apoptosis and thereby exerting antitumor effects (Figure 7A,C).

### 3.8. The Growth-Inhibitory Action of CGA-PROTAC ***A7*** on A375 Cells Is Linked to the Induction of the p53 Pathway

The p53 protein, a crucial transcription factor and tumor suppressor, governs key processes including cell cycle progression, apoptosis, and genomic integrity. Dysfunction of p53, often due to missense mutations in TP53 (encoding tumor p53), occurs in more than half of all human malignancies [30]. However, in melanoma, TP53 mutations are rare, which means alternative ways can be employed to reactivate p53-mediated tumor suppression. MDM4 shares a similar structure with MDM2 and acts as an endogenous inhibitor of p53. It exerts its inhibitory effect either by directly binding to p53 and blocking its transcriptional activity, or by forming a complex with MDM2 to facilitate MDM2-dependent p53 degradation [31]. Furthermore, MDM4 has been associated with cancer progression through mechanisms independent of p53 [32]. Gembarska et al. identified MDM4 as a crucial factor in impaired p53 function in human melanoma, highlighting it as a promising target for combination antimelanoma therapy [33]. Therefore, the impact of CGA-PROTAC **A7**-mediated MDM4 reduction on relative signaling pathways was further investigated by assessing alterations in p53 and MDM2 protein levels.

**Figure 8:**
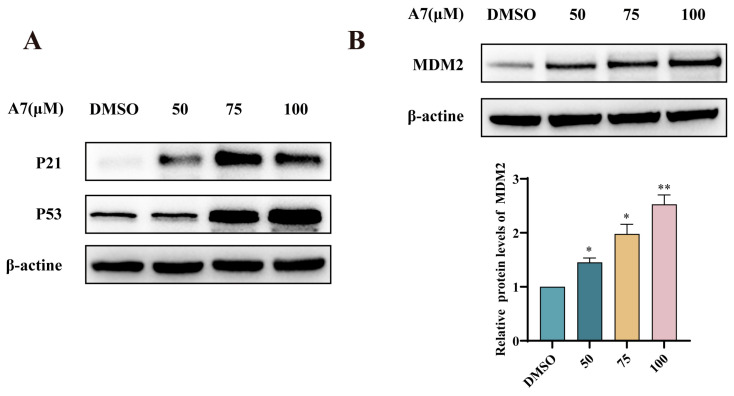
The protein expressions of P21, P53 (**A**) and MDM2 (**B**) in A375 cells following CGA-PROTAC **A7** treatment (50, 75 and 100 μM for 48 h) were measured by Western blot analysis (n = 3), using β-actine as a loading control. * *p* < 0.05, ** *p* < 0.01 compared with the negative control group (DMSO).

**Figure 9:**
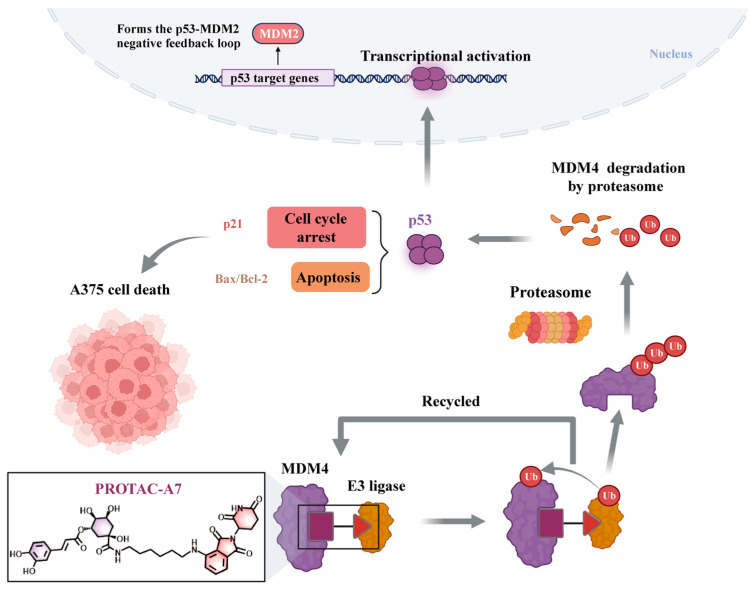
Schematic diagram of the potential mechanism of CGA-PROTAC **A7** where it degrades MDM4 protein and activates the TP53 pathway. Created in BioRender. Zhiting Mei. (2026) https://BioRender.com/hy8k4lf (accessed on 10 February 2026).

As demonstrated in Figure 8, we observed that p53 and its target gene p21 were upregulated in the cells. The expression level of MDM2 protein was significantly upregulated following CGA-PROTAC **A7** treatment, exhibiting a trend opposite to that of MDM4 protein. We speculated that upon p53 activation, activated p53 relocated to the nucleus, where it bound to the promoter region of the MDM2 gene, thereby elevating MDM2 levels (Figure 9). However, the elevated expression of p53 and p21 indicates that MDM2 may not fully suppress p53 activity. This could be attributed to the fact that MDM2 has evolved to regulate p53 transcription, nuclear export, and turnover; thus, interfering with any of these processes might be sufficient to reactivate p53. Hence, more experiments were needed to further identify the detailed mechanism.

## 4. Discussion

In summary, an integrated strategy for target identification and medication discovery of natural products was established through PROTAC derivatization and target characterization of CGA. A series of novel CGA-based PROTACs using CRBN ligase ligands were synthesized. Among these CGA derivatives, compound **A7** containing alkyl linker had a stronger inhibitory effect in the melanoma cells than CGA; the IC_50_ for A375 cells was 69.70 μM and **A7** selectively reduced MDM4 in a dose-dependent manner. CGA-PROTAC **A7** also significantly upregulated p53 and p21 protein expression, thus inhibiting proliferation. In depth research had shown that **A7** promoted the upregulation of Bax/Bcl-2, induced G2/M phase cell cycle arrest, and markedly enhanced apoptosis in melanoma A375 cells. In addition, CGA-PROTAC **A7** was synthesized in one step with an overall yield of 45.9% and provides a feasible route for the synthesis and preliminary research on the activity of the target compound and provided a basis for the combination of natural product polyphenols with PROTAC technology.

However, as an initial proof-of-concept scaffold, **A7** exhibits only modest potency but demonstrates the feasibility of this strategy. Further optimization, including linker modification, E3 ligase refinement, and CGA warhead optimization, is warranted to develop more potent derivatives. We also acknowledge that the current data only support MDM4 as a potential target of **A7**, and that the PROTAC-mediated degradation mechanism has not been fully established. Future studies will focus on elucidating the mechanism through which CGA-PROTAC **A7** induces MDM4 degradation, and on validating the binding between **A7** and MDM4 through functional direct binding assays. In vivo investigations will also be conducted to further evaluate its safety and therapeutic efficacy.

## Figures and Tables

**Table 1:** Structure of CGA-based PROTACs with purity and yield.

Compound	Linker	Purity	Yield
**A1**	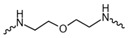	94.65%	25.12%
**A2**	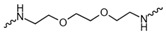	95.34%	33.75%
**A3**	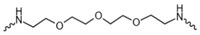	94.48%	25.45%
**A4**	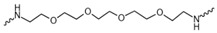	92.10%	17.13%
**A5**	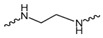	95.23%	8.94%
**A6**	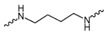	91.74%	23.82%
**A7**	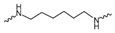	94.37%	45.96%
**A8**	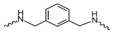	90.71%	9.85%
**A9**	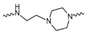	91.44%	29.13%
**A10**	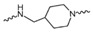	93.87%	12.52%

## Data Availability

The original contributions presented in the study are included in the article; further inquiries can be directed to the corresponding author.

## Supplementary Materials

The following supporting information can be downloaded at: https://www.mdpi.com/article/10.3390/foods15061082/s1, Figure S1: NMR spectra of compounds **A1**–**A10**; Figure S2: Purity characterization data of compounds **A1**–**A10**; Figure S3: HRMS spectra of compounds **A1**–**A10**; Table S1: Proteins significantly downregulated in proteomics.
