# Supplementary material for: Preliminary Anti-Melanoma Activity of a Chlorogenic Acid-Based PROTAC Targeting MDM4, a Candidate Protein Identified by Proteomics
Source: Foods. 2026 Mar 19;15(6):1082. doi: 10.3390/foods15061082 (PMC13025580; doi:10.3390/foods15061082)
Supplement: Supplementary file 1 [file foods-15-01082-s001.zip › foods-4175974-supplementary.pdf]

# Preliminary Anti-Melanoma Activity of a Chlorogenic Acid-Based PROTAC Targeting MDM4, a Candidate Protein Identified by Proteomics

| Contents | Page |
|---|---|
| Figure S1. NMR spectra of compounds A1-A10 | 2–16 |
| Figure S2. Purity characterization data of compounds A1-A10 | 16-22 |
| Figure S3. HRMS spectra of compounds A1-A10 | 22-27 |
| Table S1. Proteins significantly downregulated in proteomics | 27 |

# Figure S1. NMR spectra of compounds A1-A10

## 1.1 $^1$H NMR and $^1$C NMR of Compound A1 (600 MHz, DMSO-$d_6$)

(*1R,2R,3R,5S*)- 5 -( (2- (2- ((2 - (2, 6- dioxopiperidin - 3 - yl) - 1, 3- dioxoisoindolin - 4 - yl) amino) ethoxy) ethyl) carbamoyl) -2 ,3 ,5 - trihydroxycyclohexyl (*E*) - 3- (3,4-dihydroxyphenyl) acrylate. $^1$H NMR (600 MHz, DMSO-$d_6$): $\delta$ 7.74 (t, J = 5.9 Hz, 1H), 7.57 (dd, $J$ = 8.6, 7.0 Hz, 1H), 7.46 (dd, $J$ = 15.8, 1.3 Hz, 1H), 7.14 (d, $J$ = 8.6 Hz, 1H), 7.06-7.00 (m, 2H), 6.98 (dd, $J$ = 8.2, 2.1 Hz, 1H), 6.73 (d, $J$ = 8.1 Hz, 1H), 6.61 (t, $J$ = 5.9 Hz, 1H), 6.21 (dd, $J$ = 15.9, 1.0 Hz, 1H), 5.23 (td, $J$ = 10.3, 5.4 Hz, 1H), 5.05 (dd, $J$ = 12.8, 5.4 Hz, 1H), 4.10-4.05 (m, 1H), 3.59 (t, $J$ = 5.5 Hz, 2H), 3.56 (dd, $J$ = 9.7, 3.0 Hz, 1H), 3.48-3.42 (m, 4H), 3.27-3.22 (m, 2H), 2.92-2.83 (m, 1H), 2.67-2.58 (m, 1H), 2.55-2.50 (m, 1H), 2.06-1.99 (m, 1H), 1.98-1.93 (m, 1H), 1.92-1.87 (m, 1H), 1.87-1.83 (m, 1H), 1.74 (dd, $J$ = 14.1, 3.2 Hz, 1H). $^{13}$C NMR (151 MHz, DMSO): $\delta$ 174.00, 172.99, 170.22, 169.04, 167.43, 166.48, 149.44, 146.53, 145.95, 145.15, 136.39, 132.17, 125.02, 121.68, 117.56, 115.73, 114.24, 113.98, 110.82, 109.32, 76.23, 72.28, 70.98, 70.40, 68.80, 48.66, 41.78, 40.05, 38.32, 37.49, 31.07, 22.24. HRMS (ESI): m/z: [M+H]$^+$ calculated for $C_{33}H_{37}N_4O_{13}^+$ 697.2352; found: 697.2356.

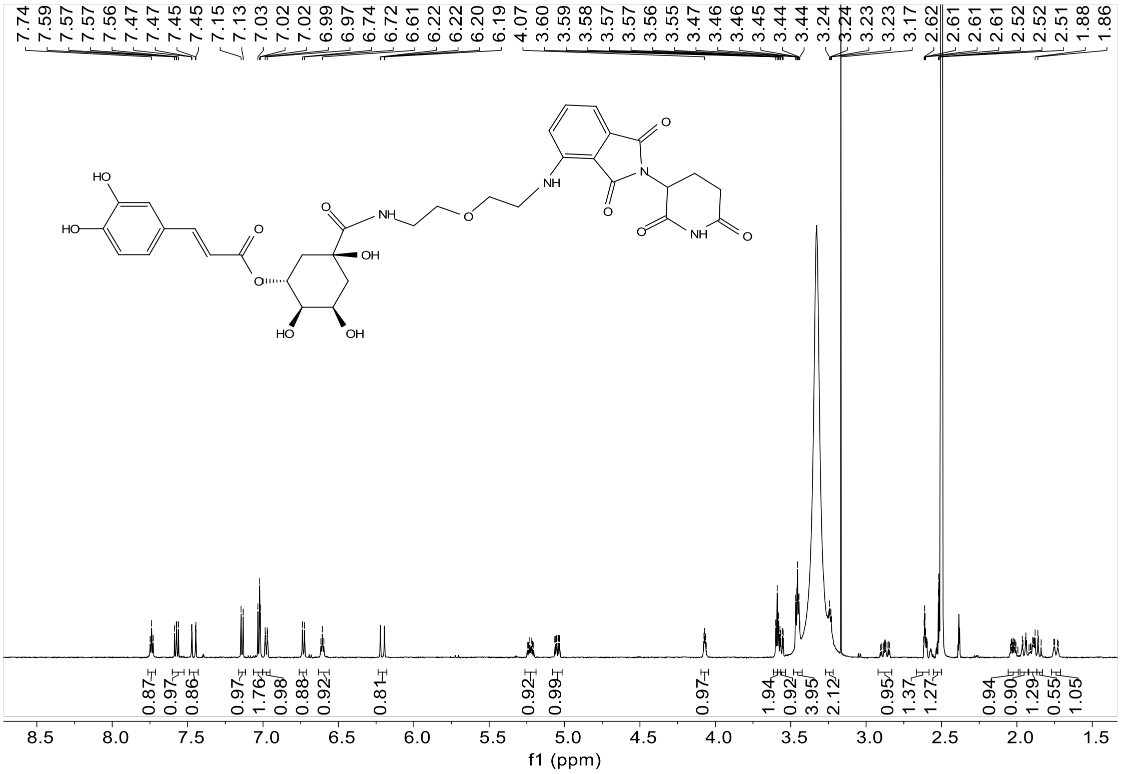

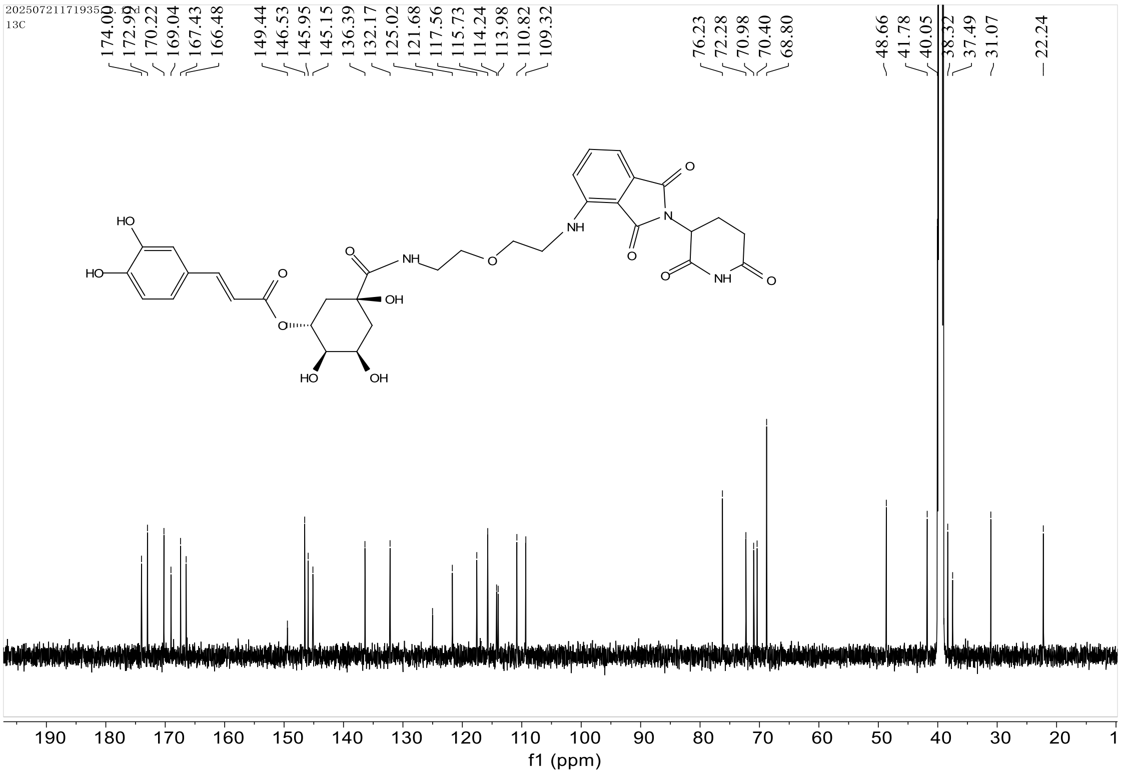


## 1.2 $^{1}$H NMR and $^{1}$C NMR of Compound A2 (600 MHz, DMSO-$d_6$)

(*1R,2R,3R,5S*)-5-( (2-( 2-(2-( (2-(2,6-dioxopiperidin -3 -yl)- 1, 3 - dioxoisoindolin-4 -yl) amino) ethoxy) ethoxy) ethyl) carbamoyl)-2,3,5-trihydroxycyclohexyl (*E*) - 3 - (3, 4-dihydroxyphenyl) acrylate. $^{1}$H NMR (600 MHz, DMSO-$d_6$): $\delta$ 7.69 (t, $J$ = 5.9 Hz, 1H), 7.58 (dd, $J$ = 8.6, 7.1 Hz, 1H), 7.46 (d, $J$ = 15.9 Hz, 1H), 7.13 (d, $J$ = 8.6 Hz, 1H), 7.03 (d, $J$ = 7.0 Hz, 1H), 7.02 (d, $J$ = 2.2 Hz, 1H), 6.97 (dd, $J$ = 8.4, 2.2 Hz, 1H), 6.73 (d, $J$ = 8.2 Hz, 1H), 6.60 (t, $J$ = 5.9 Hz, 1H), 6.21 (d, $J$ = 15.8 Hz, 1H), 5.23 (ddd, $J$ = 11.2, 9.6, 5.3 Hz, 1H), 5.05 (dd, $J$ = 12.9, 5.5 Hz, 1H), 4.08 (q, $J$ = 3.1 Hz, 1H), 3.61 (t, $J$ = 5.5 Hz, 2H), 3.59 - 3.53 (m, 3H), 3.54 - 3.49 (m, 2H), 3.49 - 3.43 (m, 2H), 3.44 - 3.39 (m, 2H), 3.27 - 3.16 (m, 2H), 2.88 (ddd, $J$ = 17.0, 13.9, 5.5 Hz, 1H), 2.62 - 2.55 (m, 1H), 2.55 - 2.52 (m, 1H), 2.03 (dtd, $J$ = 13.0, 5.3, 2.4 Hz, 1H), 1.96 (dd, $J$ = 14.5, 2.7 Hz, 1H), 1.93 - 1.82 (m, 2H), 1.77 - 1.72 (m, 1H). $^{13}$C NMR (151 MHz, DMSO): $\delta$ 173.71, 172.82, 170.11, 168.95, 167.31, 166.31, 148.98, 146.41, 145.77, 144.97, 136.24, 132.10, 125.17, 121.40, 117.44, 115.69, 114.44, 114.11, 110.67, 109.26, 76.15, 72.19, 70.90, 70.34, 69.69, 69.55, 68.90, 68.82, 48.56, 41.69, 38.26, 37.40, 30.99, 22.14. HRMS (ESI): m/z: [M+H]$^+$ calculated for $C_{35}H_{41}N_4O_{14}^+$ 741.2614; found: 741.2597.

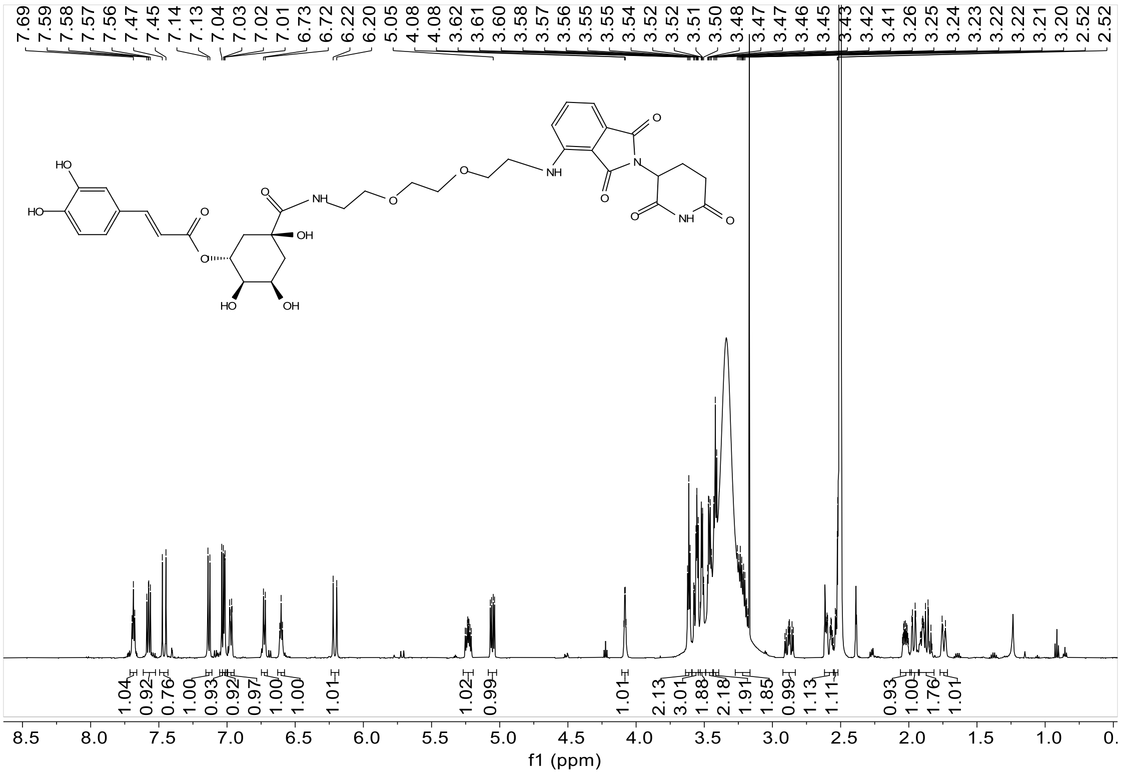


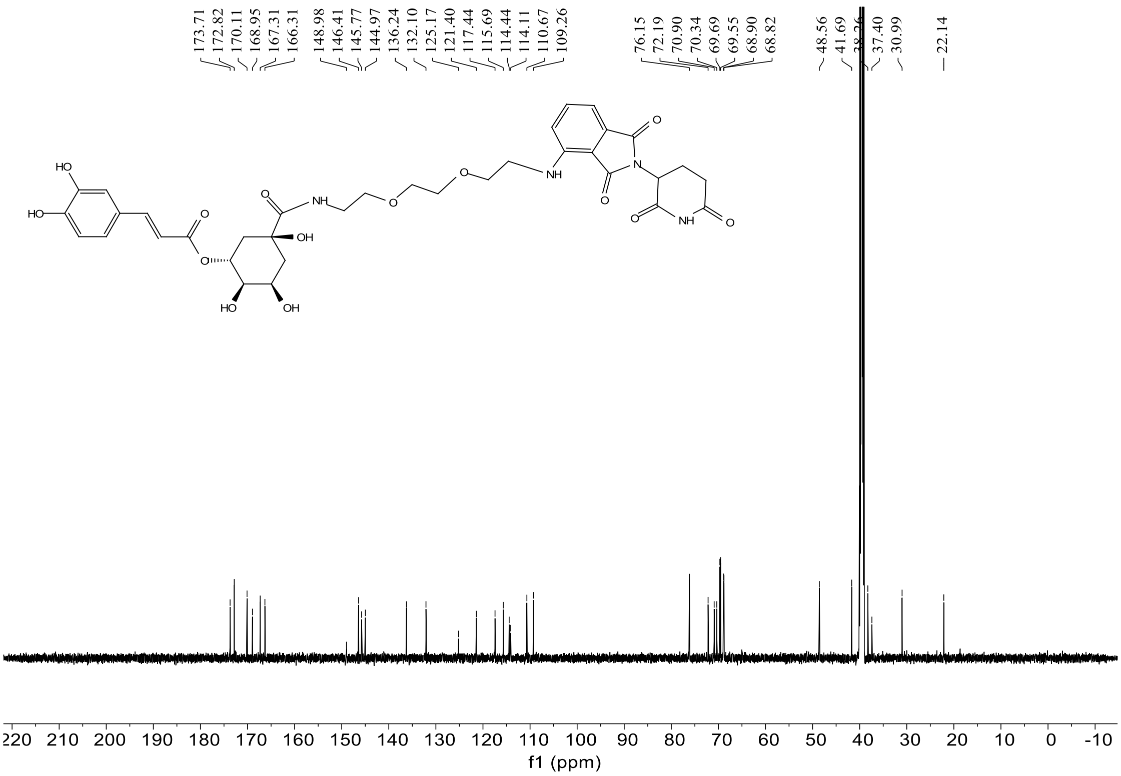


### 1.3 $^{1}$H NMR and $^{1}$C NMR of Compound A3 (600 MHz, DMSO-$d_6$)

(*1R,2R,3R,5S*)-5-( (2-( 2-(2-(2-( (2-(2,6-dioxopiperidin-3-yl)-1,3-dioxoisoindolin-4-yl) amino) ethoxy) ethoxy) ethoxy) ethyl) carbamoyl)-2,3,5-trihydroxycyclohexyl (*E*)-3-(3,4-dihydroxyphenyl) acrylate. $^{1}$H NMR (600 MHz, DMSO-$d_6$): $\delta$ 11.08 (s, 1H), 9.57 (s, 1H), 9.14 (s, 1H), 7.68 (t, $J$ = 5.9 Hz), 7.57 (t, $J$ = 7.8 Hz, 1H), 7.47 (d, $J$ = 15.8

Hz, 1H), 7.13 (d, $J$ = 8.6 Hz, 1H), 7.03 (m, 2H), 6.99 (dd, $J$ = 8.0, 2.1 Hz, 1H), 6.76 (d, $J$ = 8.1 Hz, 1H), 6.59 (t, $J$ = 5.9 Hz, 1H), 6.23 (d, $J$ = 15.8 Hz, 1H), 5.70 (d, $J$ = 3.9 Hz, 1H), 5.60 (s, 1H), 5.23 (m, 1H), 5.05 (m, 2H), 4.08 (d, $J$ = 3.5 Hz, 1H), 3.61 (t, $J$ = 5.4 Hz, 2H), 3.56 (m, 2H), 3.49 (m, 10H), 3.39 (t, $J$ = 6.0 Hz, 2H), 3.20 (m, 2H), 2.88 (m, 1H), 2.59 (m, 1H), 2.02 (m, 1H), 1.96 (m, 1H), 1.86 (m, 1H), 1.74 (m, 1H). $^{13}C$ NMR (151 MHz, DMSO): $\delta$ 173.81, 172.91, 170.16, 169.00, 167.38, 166.38, 148.83, 146.47, 145.79, 145.01, 136.31, 132.14, 125.36, 121.45, 117.52, 115.80, 114.55, 114.28, 110.75, 109.28, 76.21, 72.25, 70.95, 70.40, 69.86, 69.83, 69.58, 68.93, 68.79, 48.61, 41.75, 40.05, 38.32, 37.45, 31.03, 22.20. HRMS (ESI): m/z: $[M+H]^+$ calculated for $C_{37}H_{45}N_4O_{15}^+$ 785.2876; found: 785.2870.

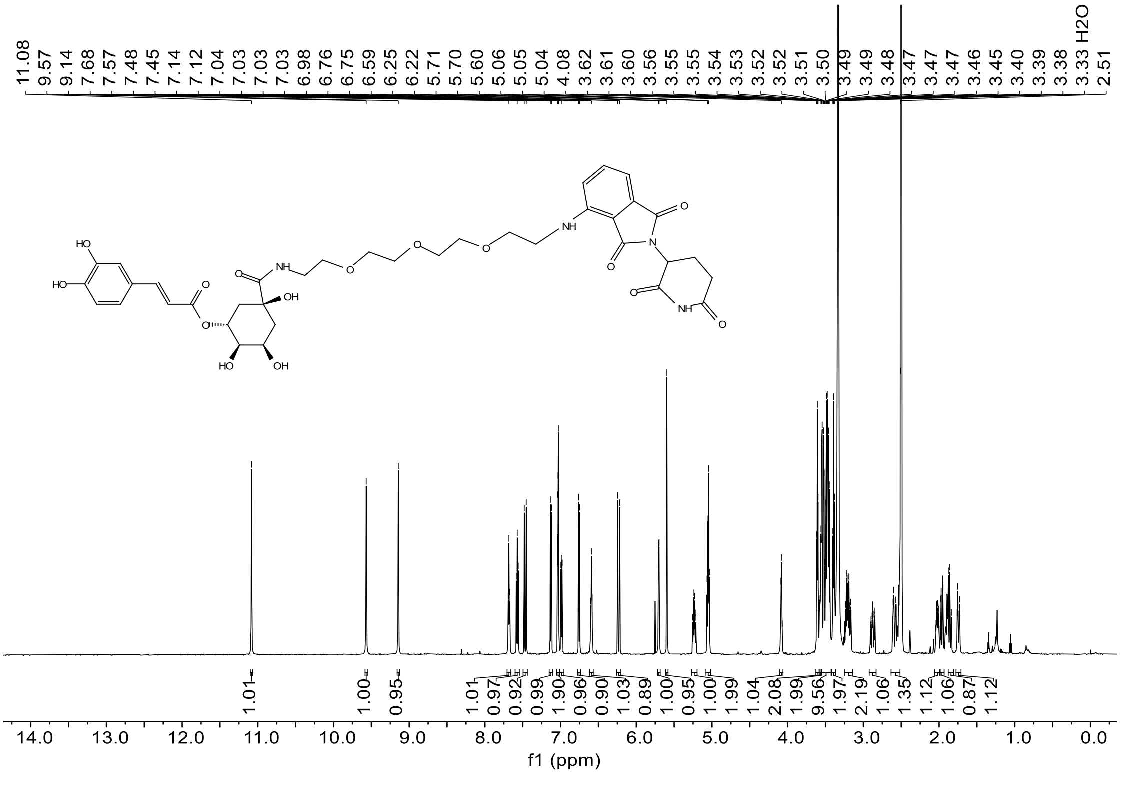

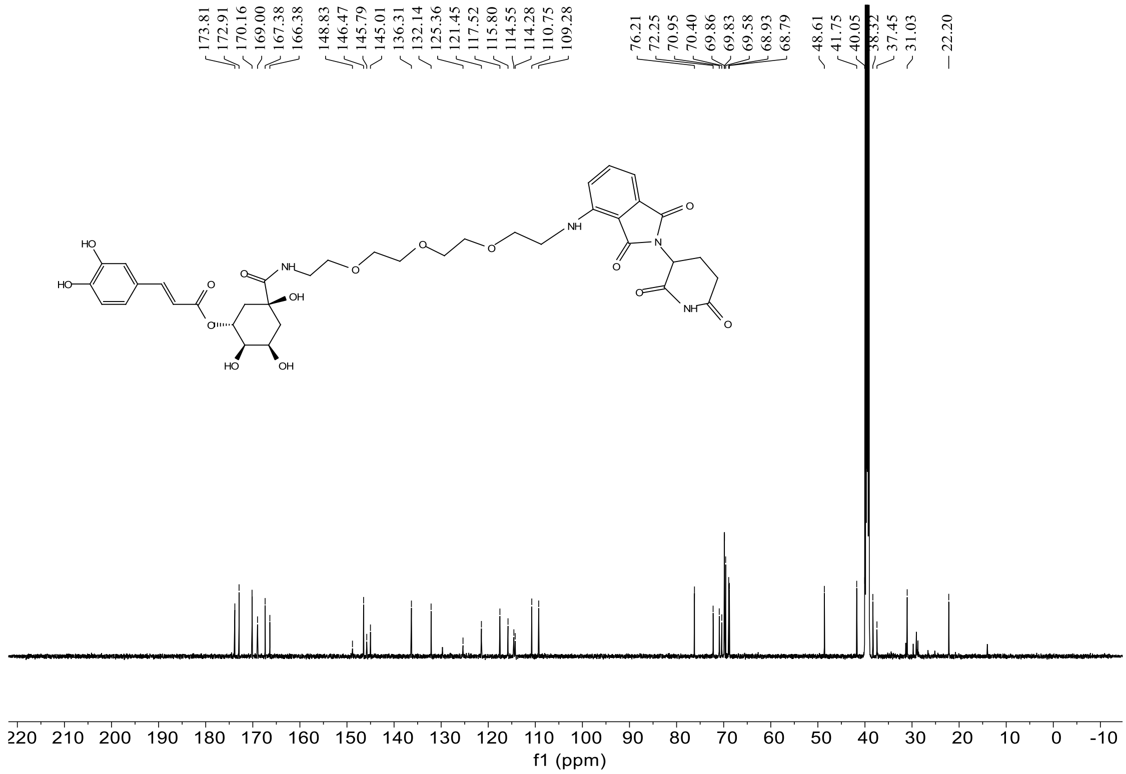


## 1.4 $^{1}$H NMR and [1]C NMR of Compound A4 (600 MHz, DMSO-$d_6$)

(*1R,2R,3R,5S*) – 5 - ((14-( (2 - (2, 6- dioxopiperidin – 3 - yl)- 1, 3 – dioxoisoindolin - 4 -yl) amino)- 3, 6, 9, 12 - tetraoxatetradecyl) carbamoyl) -2, 3,5-trihydroxycyclohexyl (*E*)-3-(3,4-dihydroxyphenyl) acrylate. $^1$H NMR (600 MHz, DMSO-$d_6$): $\delta$ 7.69 (t, $J$ = 5.9 Hz, 1H), 7.58 (dd, $J$ = 8.5, 7.0 Hz, 1H), 7.47 (d, $J$ = 15.9 Hz, 1H), 7.14 (d, $J$ = 8.6 Hz, 1H), 7.07 - 7.01 (m, 2H), 6.99 (dd, $J$ = 8.2, 2.2 Hz, 1H), 6.75 (d, $J$ = 8.1 Hz, 1H), 6.60 (t, $J$ = 5.9 Hz, 1H), 6.23 (d, $J$ = 15.9 Hz, 1H), 5.23 (ddd, $J$ = 11.1, 9.6, 5.3 Hz, 1H), 5.05 (dd, $J$ = 12.8, 5.4 Hz, 1H), 4.08 (q, $J$ = 3.1 Hz, 1H), 3.61 (t, $J$ = 5.5 Hz, 2H), 3.59 - 3.53 (m, 3H), 3.53 - 3.49 (m, 2H), 3.50 - 3.47 (m, 2H), 3.49 - 3.48 (m, 2H), 3.47 - 3.44 (m, 6H), 3.39 (t, $J$ = 6.1 Hz, 2H), 3.24 - 3.18 (m, 1H), 2.92 - 2.83 (m, 1H), 2.63 - 2.50 (m, 2H), 2.06 - 1.98 (m, 1H), 1.99 - 1.93 (m, 1H), 1.95 - 1.87 (m, 1H), 1.89 - 1.82 (m, 1H), 1.78 - 1.71 (m, 1H). $^{13}$C NMR (151 MHz, DMSO): $\delta$ 173.71, 172.83, 170.09, 168.95, 167.31, 166.26, 148.44, 146.43, 145.62, 144.88, 136.24, 132.10, 125.52, 121.29, 117.47, 115.76, 114.68, 114.43, 110.68, 109.25, 76.15, 72.19, 70.90, 70.37, 69.84, 69.80, 69.77, 69.70, 69.52, 68.88, 68.76, 48.57, 41.71, 38.26, 37.42, 30.99, 22.15. HRMS (ESI): m/z: $[M+H]^+$ calculated for $C_{39}H_{49}N_4O_{16}^+$ 829.3138; found: 829.3158

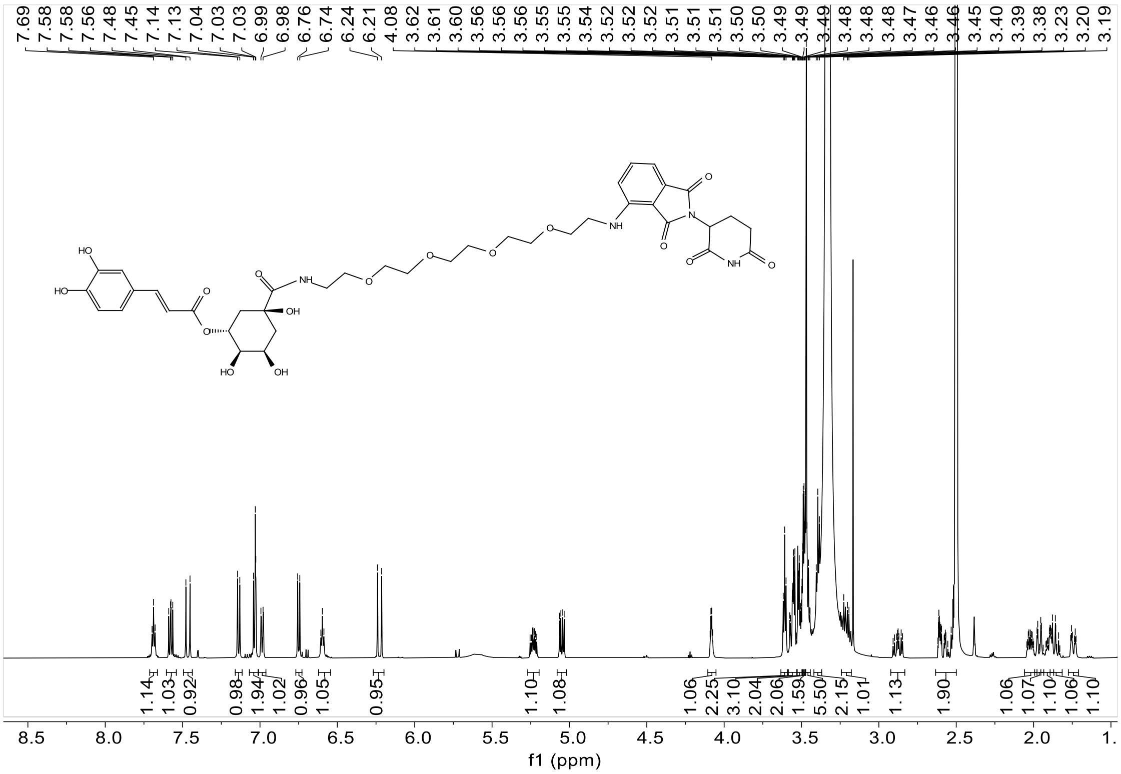

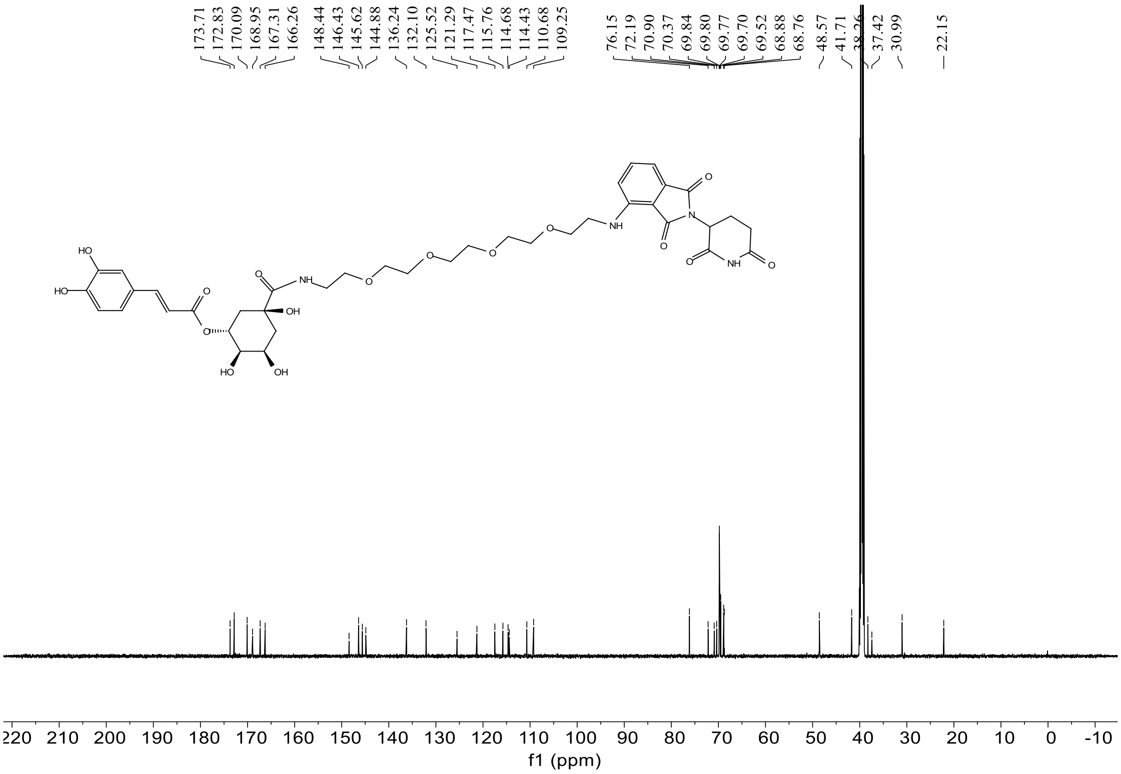

## 1.5 $^{1}$H NMR and $^{1}$C NMR of Compound A5 (600 MHz, DMSO-$d_6$)

(*1R,2R,3R,5S*)- 5 -( (2 - ((2 - (2, 6 – dioxopiperidin - 3 - yl) - 1, 3 - dioxoisoindolin - 4 - yl) amino) ethyl) carbamoyl) -2, 3, 5- trihydroxycyclohexyl (*E*) -3- (3, 4 - dihydroxyphenyl) acrylate. $^{1}$H NMR (600 MHz, DMSO-$d_6$): $\delta$ 11.08 (s, 1H), 8.08 (t, $J$ = 5.9 Hz, 1H), 7.56 (dd, $J$ = 8.6, 7.1 Hz, 1H), 7.46 (d, $J$ = 15.8 Hz, 1H), 7.21 (dd, $J$ =

8.6, 1.5 Hz, 1H), 7.03 (d, *J* = 2.1 Hz, 1H), 7.00 (d, *J* = 7.1 Hz, 1H), 6.99 (dd, *J* = 8.2, 2.1 Hz, 1H), 6.75 (d, *J* = 8.1 Hz, 1H), 6.71 (t, *J* = 6.1 Hz, 1H), 6.22 (d, *J* = 15.9 Hz, 1H), 5.26 - 5.19 (m, 1H), 5.04 (dd, *J* = 12.8, 5.4 Hz, 1H), 4.09 - 4.05 (m, 1H), 3.56 (dd, *J* = 9.6, 2.9 Hz, 1H), 3.42 - 3.20 (m, 4H), 2.91 - 2.82 (m, 1H), 2.63 - 2.54 (m, 2H), 2.03 - 1.98 (m, 1H), 1.98 - 1.92 (m, 1H), 1.91 - 1.81 (m, 2H), 1.76 - 1.70 (m, 1H). $^{13}$C NMR (151 MHz, DMSO): $\delta$ 174.36, 172.82, 170.10, 168.68, 167.32, 166.33, 148.41, 146.41, 145.57, 144.55, 136.17, 132.20, 123.99, 121.42, 117.33, 115.71, 114.86, 113.53, 110.52, 109.21, 76.18, 72.21, 70.90, 70.33, 48.52, 41.33, 37.87, 37.44, 30.98, 22.16. HRMS(ESI): m/z: $[M+H]^+$ calculated for $C_{31}H_{33}N_4O_{12}^+$ 653.2089; found: 653.2080.

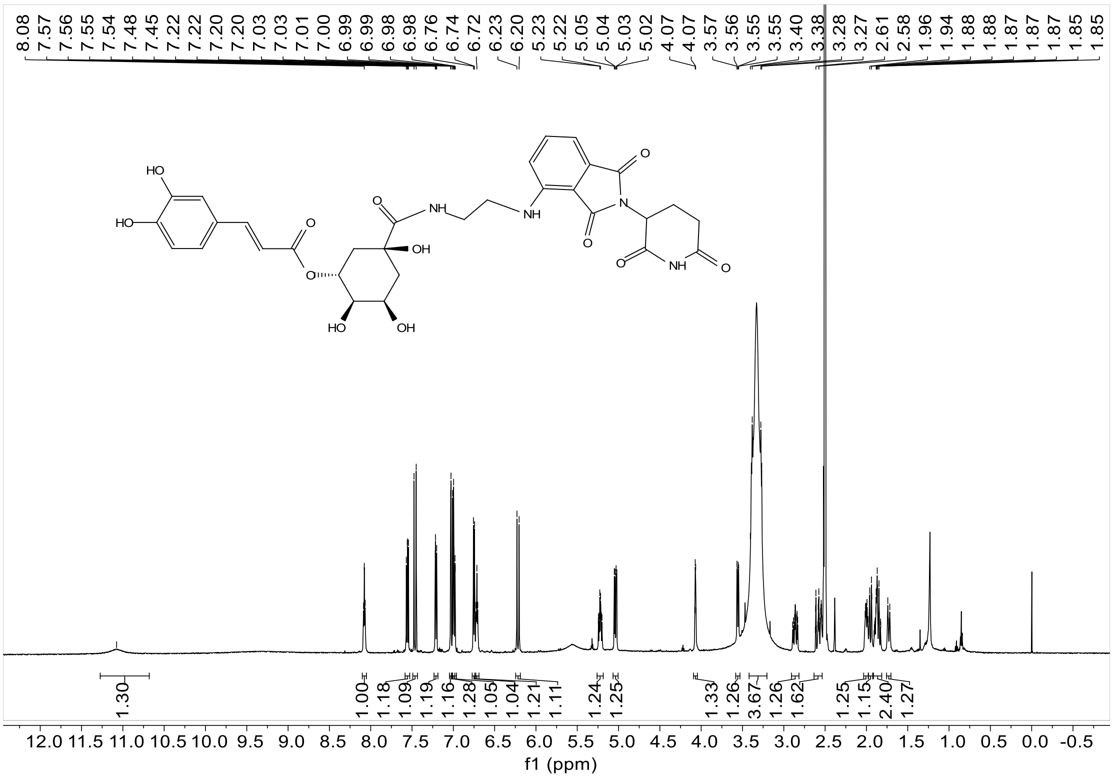

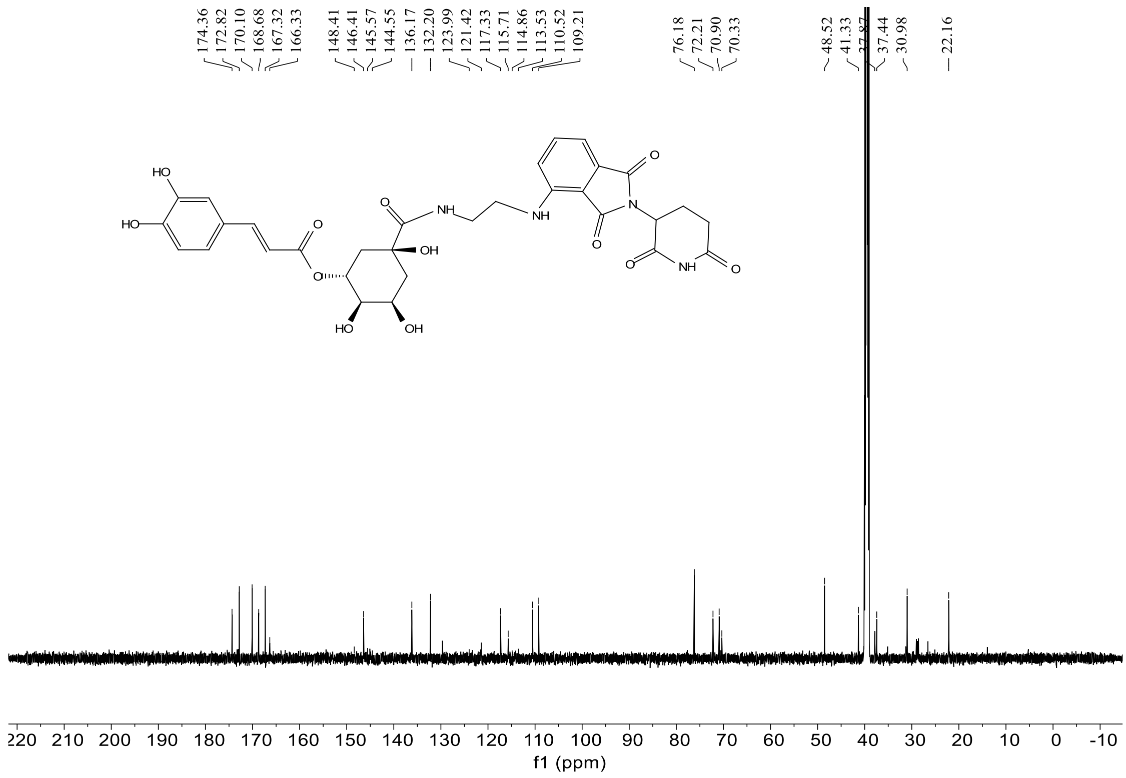


## 1.6 $^{1}$H NMR and $^{1}$C NMR of Compound A6 (600 MHz, DMSO-$d_6$)

(*1R,2R,3R,5S*)- 5 -( (4 -( (2 - (2, 6- dioxopiperidin - 3 - yl) -1, 3- dioxoisoindolin - 4 - yl) amino) butyl) carbamoyl) -2,3,5- trihydroxycyclohexyl (*E*) - 3 - (3, 4- dihydroxyphenyl) acrylate. $^{1}$H NMR (600 MHz, DMSO-$d_6$): $\delta$ 11.08 (s, 1H), 9.57 (s, 1H), 9.14 (s, 1H), 7.83 (t, $J$ = 6.2 Hz, 1H), 7.55 (t, $J$ = 7.8 Hz, 1H), 7.47 (d, $J$ = 15.9 Hz, 1H), 7.07 (d, $J$ = 8.6 Hz, 1H), 7.04 (s, 1H), 6.99 (d, $J$ = 7.0 Hz, 2H), 6.76 (d, $J$ = 8.1 Hz, 1H), 6.56 (t, $J$ = 6.1 Hz, 1H), 6.24 (d, $J$ = 15.9 Hz, 1H), 5.64 (d, $J$ = 3.9 Hz, 1H), 5.57 (s, 1H), 5.24 (td, $J$ = 9.9, 6.0 Hz, 1H), 5.04 (dd, $J$ = 12.1, 5.5 Hz, 2H), 4.13 - 3.98 (m, 1H), 3.59 - 3.52 (m, 1H), 3.30 (m, 1H), 3.29 (d, $J$ = 6.0 Hz, 2H), 3.10 (hept, $J$ = 6.6 Hz, 2H), 2.87 (ddd, $J$ = 18.1, 13.8, 5.4 Hz, 1H), 2.67 - 2.54 (m, 2H), 2.05 - 1.93 (m, 3H), 1.89 (d, $J$= 10.5 Hz, 2H), 1.61 - 1.43 (m, 4H). $^{13}$C NMR (151 MHz, DMSO)： $\delta$ 173.69, 172.87, 170.14, 168.93, 167.34, 166.29, 148.43, 146.41, 145.64, 144.90, 136.27, 132.23, 125.57, 121.31, 117.24, 115.81, 114.71, 114.48, 110.37, 109.03, 76.15, 72.22, 70.89 , 70.44, 48.56, 41.54, 40.05, 37.96, 37.54, 31.00, 26.49, 25.96, 22.18. HRMS (ESI): m/z: $[M+H]^+$ calculated for $C_{33}H_{37}N_4O_{12}^+$ 681.2402; found: 681.2425.

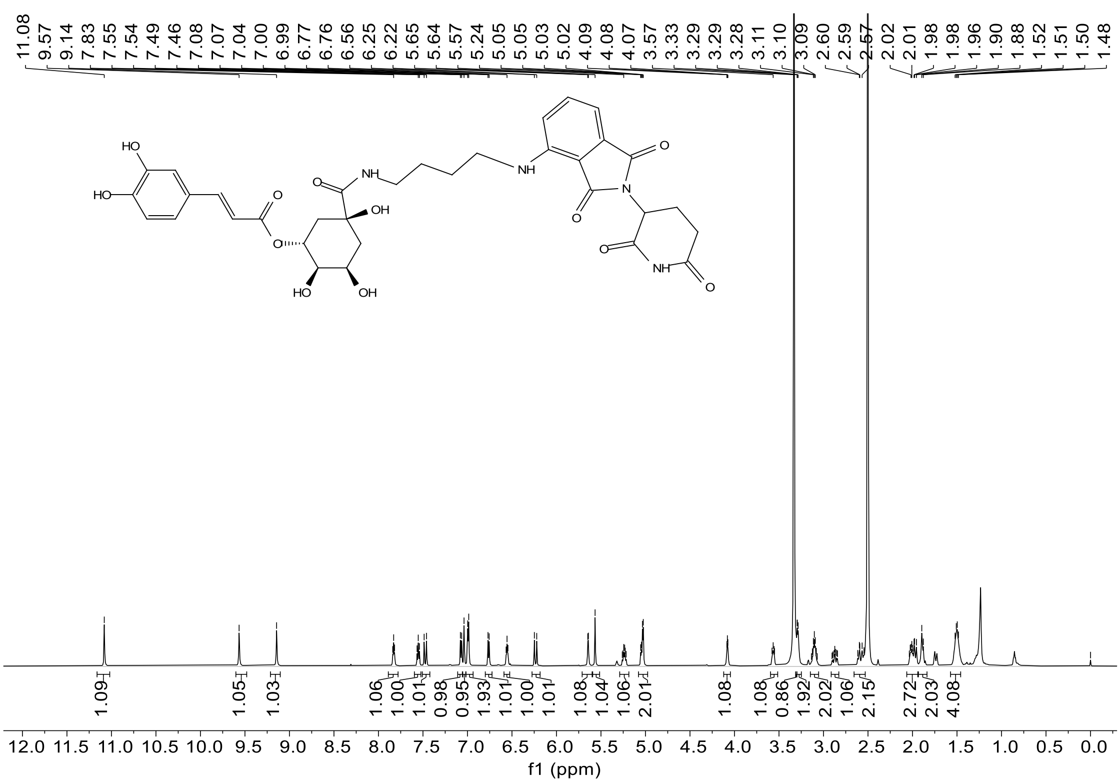


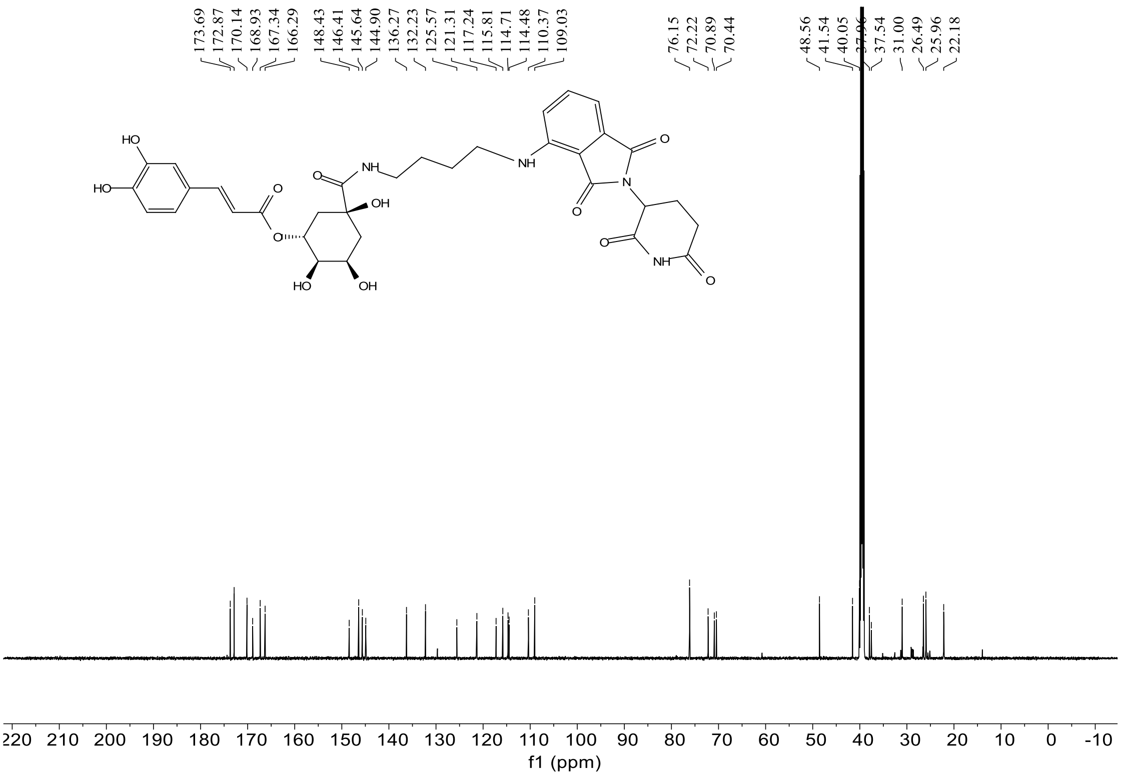


## 1.7 $^{1}$H NMR and $^{1}$C NMR of Compound A7 (600 MHz, DMSO-$d_6$)

(*1R,2R,3R,5S*)- 5 -( (6 -((2- (2,6-dioxopiperidin- 3 -yl) -1, 3- dioxoisoindolin-4-yl) amino) hexyl) carbamoyl) -2, 3, 5-trihydroxycyclohexyl (*E*) - 3-(3, 4-dihydroxyphenyl) acrylate. $^{1}$H NMR (600 MHz, DMSO-$d_6$): $\delta$ 11.08 (s, 1H), 9.56 (s, 1H), 9.14 (s, 1H), 7.73 (t, $J$ = 6.0 Hz, 1H), 7.57 (dd, $J$ = 8.6, 7.0 Hz, 1H), 7.47 (d, $J$ = 15.9 Hz, 1H), 7.07

(d, $J$ = 8.7 Hz, 1H), 7.03 (d, $J$ = 2.1 Hz, 1H), 7.01 (d, $J$ = 7.0 Hz, 1H), 6.99 (dd, $J$ = 8.3, 2.2 Hz, 1H), 6.76 (d, $J$ = 8.2 Hz, 1H), 6.52 (t, $J$ = 5.9 Hz, 1H), 6.23 (d, $J$ = 15.9 Hz, 1H), 5.64 (d, $J$ = 4.2 Hz, 1H), 5.55 (s, 1H), 5.23 (td, $J$ = 10.0, 5.9 Hz, 1H), 5.07 - 5.01 (m, 2H), 4.11 - 4.05 (m, 1H), 3.56 (ddd, $J$ = 9.2, 5.8, 2.9 Hz, 1H), 3.26 (t, $J$ = 6.8 Hz, 2H), 3.17 (d, $J$ = 5.2 Hz, 1H), 3.05 (dq, $J$ = 12.7, 6.4 Hz, 2H), 2.92 - 2.83 (m, 1H), 2.65 - 2.51 (m, 2H), 2.02 (dtd, $J$ = 13.1, 5.4, 2.4 Hz, 1H), 1.96 (d, $J$ = 13.4 Hz, 1H), 1.93 - 1.82 (m, 2H), 1.76 - 1.71 (m, 1H), 1.55 (p, $J$ = 7.4 Hz, 2H), 1.41 (p, $J$ = 7.2 Hz, 2H), 1.36 - 1.22 (m, 4H). $^{13}$C NMR (151 MHz, DMSO): $\delta$ 173.51, 172.84, 170.13, 168.95, 167.33, 166.26, 148.42, 146.43, 145.61, 144.87, 136.30, 132.21, 125.54, 121.28, 117.18, 115.77, 114.69, 114.45, 110.38, 109.03, 76.10, 72.18, 70.85, 70.44, 48.55, 41.78, 38.29, 37.54, 30.99, 29.03, 28.63, 26.00, 25.97, 22.16. HRMS (ESI): m/z: $[M+H]^+$ calculated for $C_{35}H_{41}N_4O_{12}^+$ 709.2715; found: 709.2726.

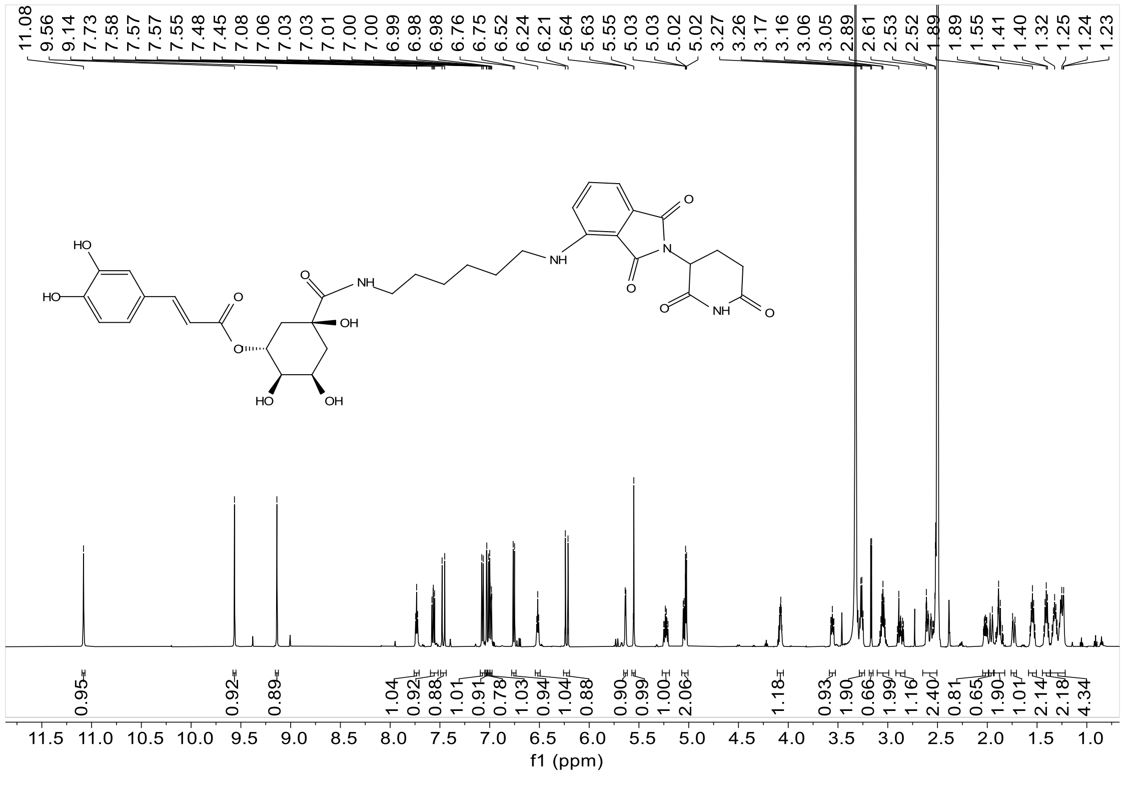

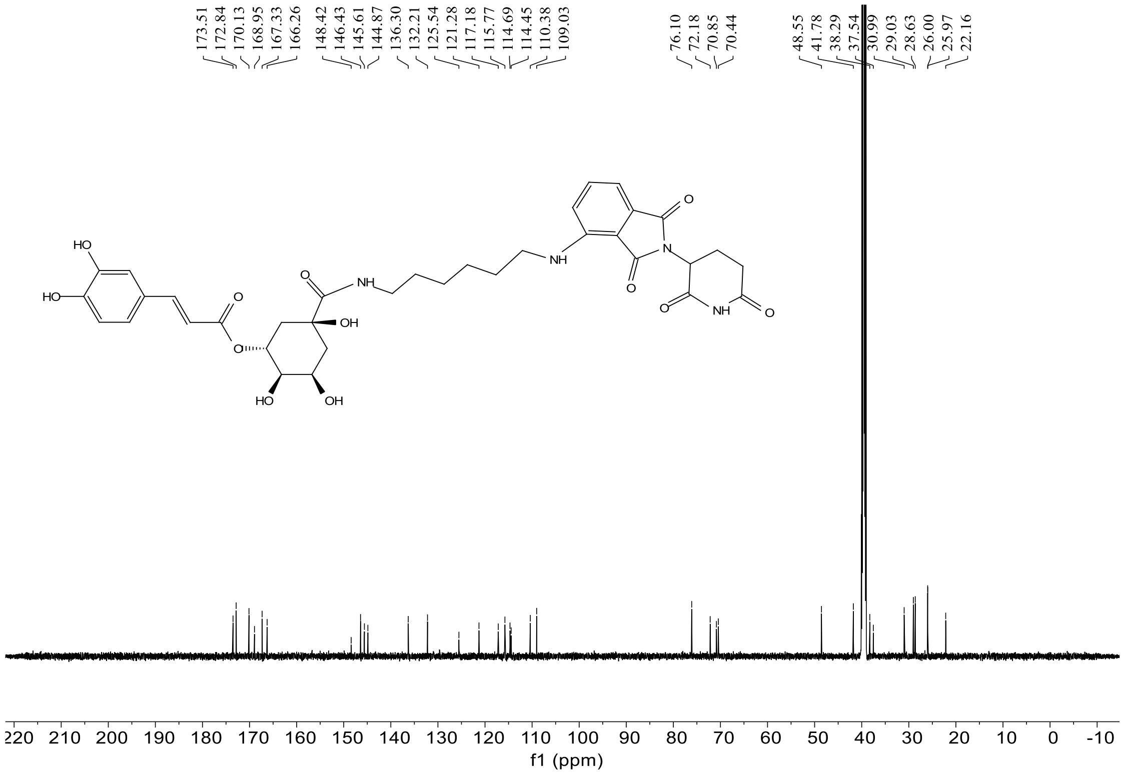


## 1.8 $^1$H NMR and $^1$C NMR of Compound A8 (600 MHz, DMSO-$d_6$)

*(1R,2R,3R,5S)*- 5 -( (3 -( ((2 -(2, 6- dioxopiperidin- 3 - yl) -1, 3- dioxoisoindolin - 4-yl) amino) methyl) benzyl) carbamoyl)-2, 3, 5 - trihydroxycyclohexyl (*E*) - 3 -(3, 4- dihydroxyphenyl) acrylate. $^1$H NMR (600 MHz, DMSO-$d_6$): $\delta$ 8.32 (t, $J$ = 6.4 Hz, 1H), 7.49 (dd, $J$ = 8.6, 7.1 Hz, 1H), 7.45 (d, $J$ = 15.8 Hz, 1H), 7.27 (t, $J$ = 7.5 Hz, 1H), 7.24 - 7.20 (m, 2H), 7.22 - 7.16 (m, 1H), 7.10 (d, $J$ = 7.6 Hz, 1H), 7.01 (d, $J$ = 7.1 Hz, 1H), 6.99 (d, $J$ = 2.1 Hz, 1H), 6.94 (td, $J$ = 8.4, 2.3 Hz, 2H), 6.67 (d, $J$ = 8.0 Hz, 1H), 6.17 (d, $J$ = 15.8 Hz, 1H), 5.28 - 5.21 (m, 1H), 5.10 - 5.04 (m, 1H), 4.53 (d, $J$ = 6.2 Hz, 2H), 4.26 - 4.19 (m, 2H), 4.09 - 4.06 (m, 1H), 3.59 - 3.54 (m, 1H), 2.93 - 2.84 (m, 1H), 2.63 - 2.60 (m, 2H), 2.07 - 2.02 (m, 1H), 1.98 - 1.92 (m, 1H), 1.92 - 1.85 (m, 2H), 1.79 - 1.72 (m, 1H). $^{13}$C NMR (151 MHz, DMSO): $\delta$ 173.78, 172.84, 170.12, 168.76, 167.29, 166.26, 148.37, 146.11, 145.60, 144.88, 140.04, 138.95, 136.10, 132.19, 128.47, 127.15, 125.64, 125.53, 125.23, 121.24, 117.57, 115.81, 114.75, 114.44, 110.73, 109.51, 76.19, 72.25, 70.91, 70.39, 48.57, 45.52, 41.86, 37.51, 30.99, 22.15. HRMS (ESI): m/z: $[M+H]^+$ calculated for $C_{37}H_{37}N_4O_{12}^+$ 729.2402; found: 729.2387.

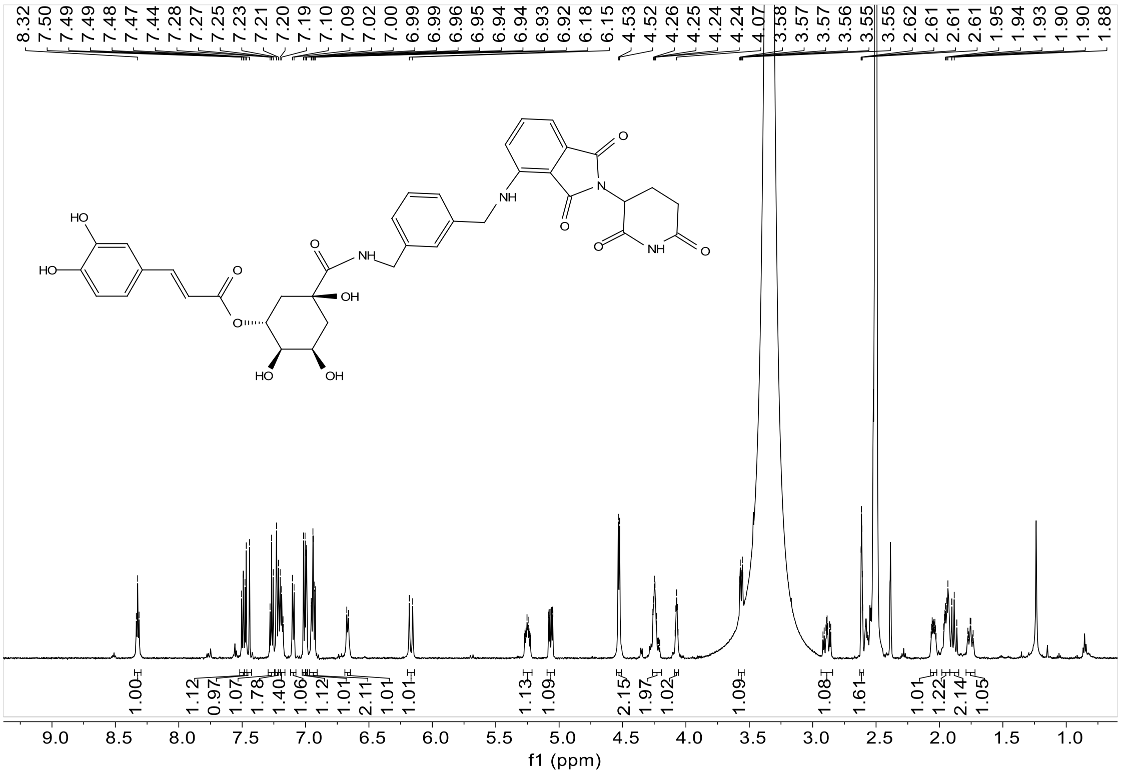


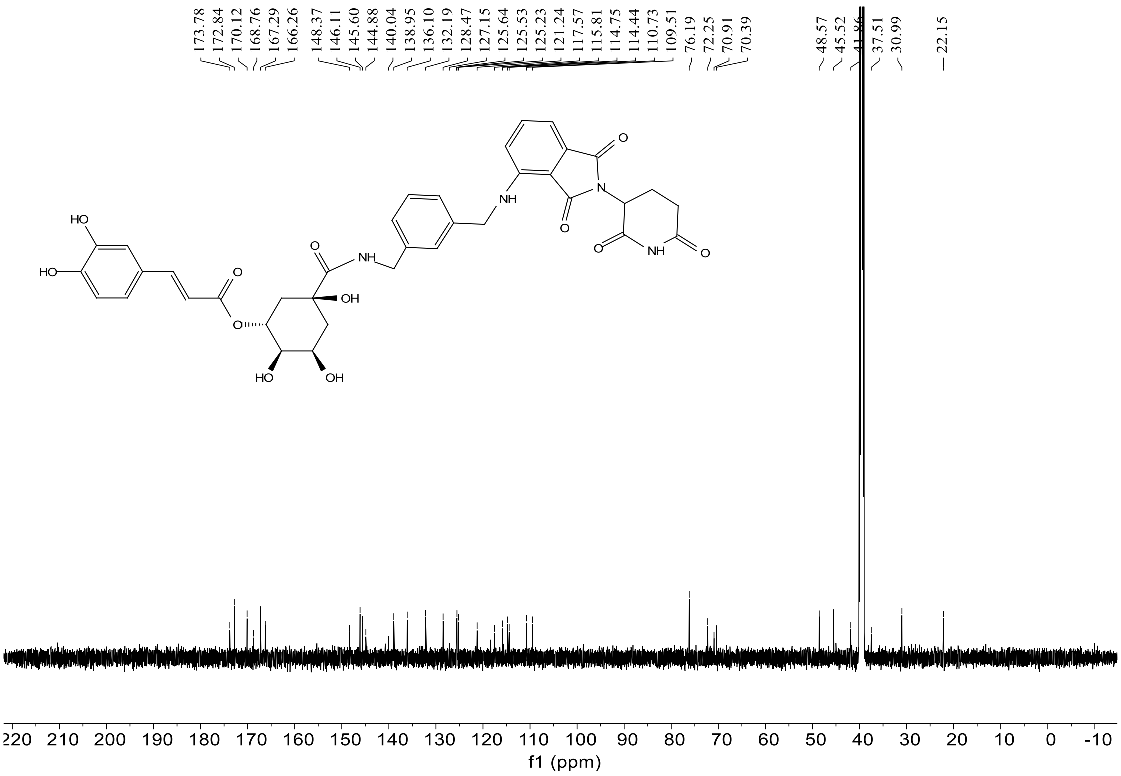


## 1.9 $^{1}$H NMR and $^{1}$C NMR of Compound A9 (600 MHz, DMSO-$d_6$)

*(1R,2R,3R,5S)*- 5 -( (2 - (4 - (2- (2, 6 - dioxopiperidin - 3 -yl) - 1, 3 - dioxoisoindolin -4 - yl) piperazin - 1 -yl) ethyl) carbamoyl) - 2, 3, 5 - trihydroxycyclohexyl (*E*) -3 -(3, 4-dihydroxyphenyl) acrylate. $^{1}$H NMR (600 MHz, DMSO-$d_6$): $\delta$ 7.72 (t, $J$ = 5.8 Hz, 1H), 7.68 (dd, $J$ = 8.4, 7.1 Hz, 1H), 7.47 (d, $J$ = 15.8 Hz, 1H), 7.36 - 7.32 (m, 1H), 7.34

- 7.27 (m, 1H), 7.04 (d, $J$ = 2.1 Hz, 1H), 6.98 (dd, $J$ = 8.3, 1.8 Hz, 1H), 6.75 (d, $J$ = 8.1 Hz, 1H), 6.22 (d, $J$ = 16.0 Hz, 1H), 5.24 (ddd, $J$ = 11.1, 9.7, 5.2 Hz, 1H), 5.09 (dd, $J$ = 12.9, 5.5 Hz, 1H), 4.10 (q, $J$ = 3.1 Hz, 1H), 3.58 (dd, $J$ = 9.6, 2.9 Hz, 1H), 3.26 - 3.21 (m, 6H), 2.87 (ddd, $J$ = 17.0, 13.9, 5.5 Hz, 1H), 2.62 - 2.52 (m, 4H), 2.47 - 2.42 (m, 2H), 2.06 - 1.80 (m, 4H), 1.80 - 1.74 (m, 1H). $^{13}$C NMR (151 MHz, DMSO): $\delta$ 173.60, 172.82, 170.02, 167.06, 166.33, 166.30, 149.70, 149.49, 145.88, 145.01, 135.87, 133.66, 123.73, 121.42, 117.53, 116.52, 115.71, 114.80, 114.40, 114.00, 76.17, 72.21, 70.90, 70.39, 56.61, 52.50, 51.23, 50.53, 48.80, 37.45, 35.74, 30.96, 22.05. HRMS (ESI): m/z: [M+H]$^+$ calculated for $C_{35}H_{40}N_5O_{12}^+$ 722.2668; found: 722.2649.

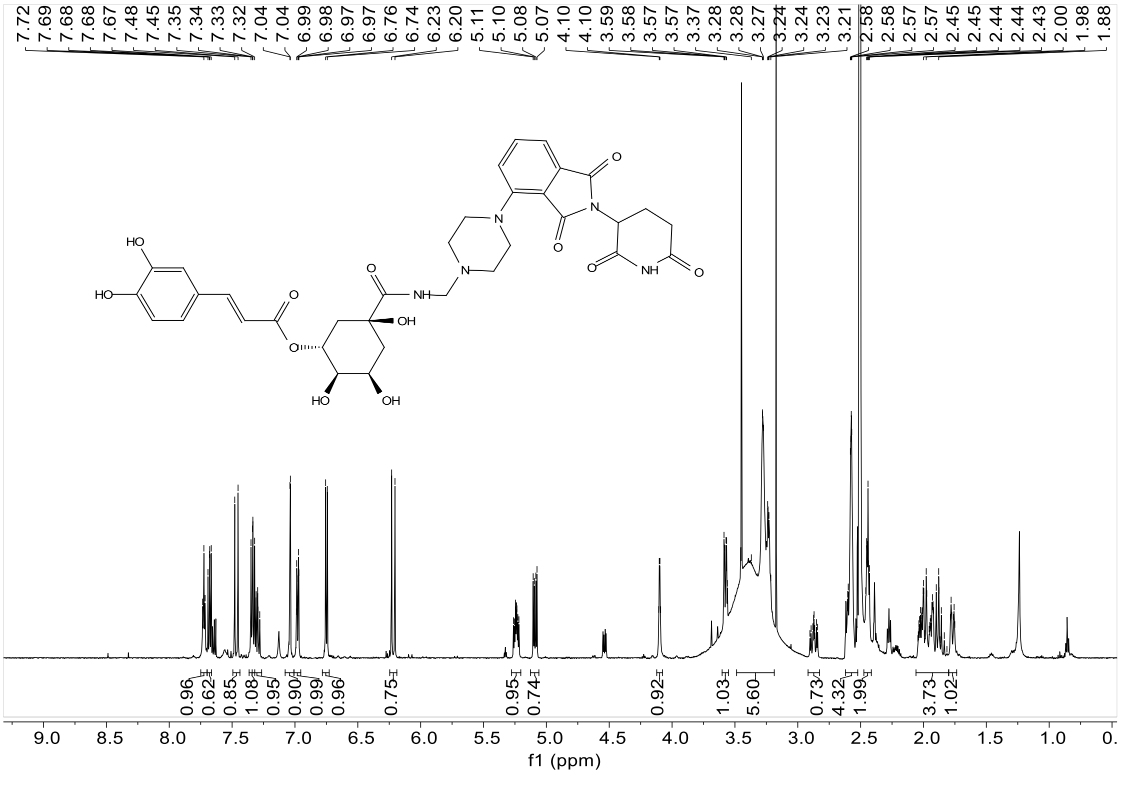

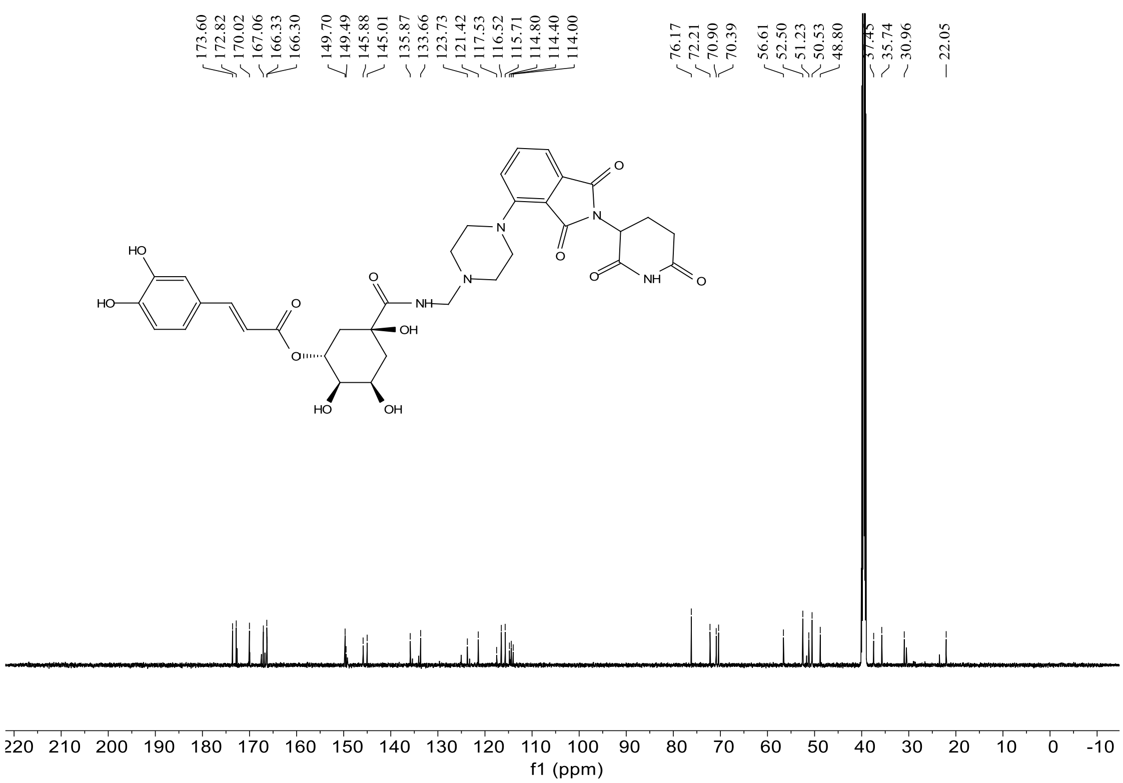


## 1.10 $^{1}$H NMR and $^{1}$C NMR of Compound A10 (600 MHz, DMSO-$d_6$)

*(1R,2R,3R,5S)* - 5 - (((1- (2- (2, 6 – dioxopiperidin - 3-yl) -1, 3- dioxoisoindolin - 4 -yl) piperidin- 4 -yl) methyl) carbamoyl) -2, 3, 5 - trihydroxycyclohexyl (*E*)-3-(3,4-dihydroxyphenyl) acrylate. $^{1}$H NMR (600 MHz, DMSO-$d_6$): $\delta$ 7.91 - 7.79 (m, 1H), 7.65 (t, $J$ = 7.9 Hz, 1H), 7.44 (d, $J$ = 15.8 Hz, 1H), 7.30 (d, $J$ = 7.8 Hz, 2H), 6.98 (s, 1H), 6.91 (d, $J$ = 8.1 Hz, 1H), 6.65 (d, $J$ = 7.9 Hz, 1H), 6.15 (d, $J$ = 16.0 Hz, 1H), 5.23 (q, $J$ = 8.7 Hz, 1H), 5.06 (dd, $J$ = 12.6, 5.9 Hz, 1H), 4.10 - 4.07 (m, 1H), 3.84 - 3.61 (m, 3H), 3.11 - 2.97 (m, 2H), 2.90 - 2.74 (m, 4H), 2.58 (d, $J$ = 17.5 Hz, 1H), 2.03 - 1.96 (m, 2H), 1.95 - 1.85 (m, 2H), 1.79 - 1.74 (m, 1H), 1.71 - 1.62 (m, 4H). $^{13}$C NMR (151 MHz, DMSO): $\delta$ 173.76, 172.79, 170.04, 167.10, 166.33, 166.28, 150.13, 149.40, 145.93, 145.07, 135.70, 133.66, 123.93, 121.51, 116.34, 115.60, 114.37, 114.18, 113.86, 113.77, 76.18, 72.18, 70.84, 70.34, 50.84, 48.76, 43.72, 40.06, 37.61, 35.24, 30.95, 29.51, 22.06. HRMS (ESI): m/z: $[M+H]^+$ calculated for $C_{35}H_{39}N_4O_{12}^+$ 707.2559; found: 707.2548.

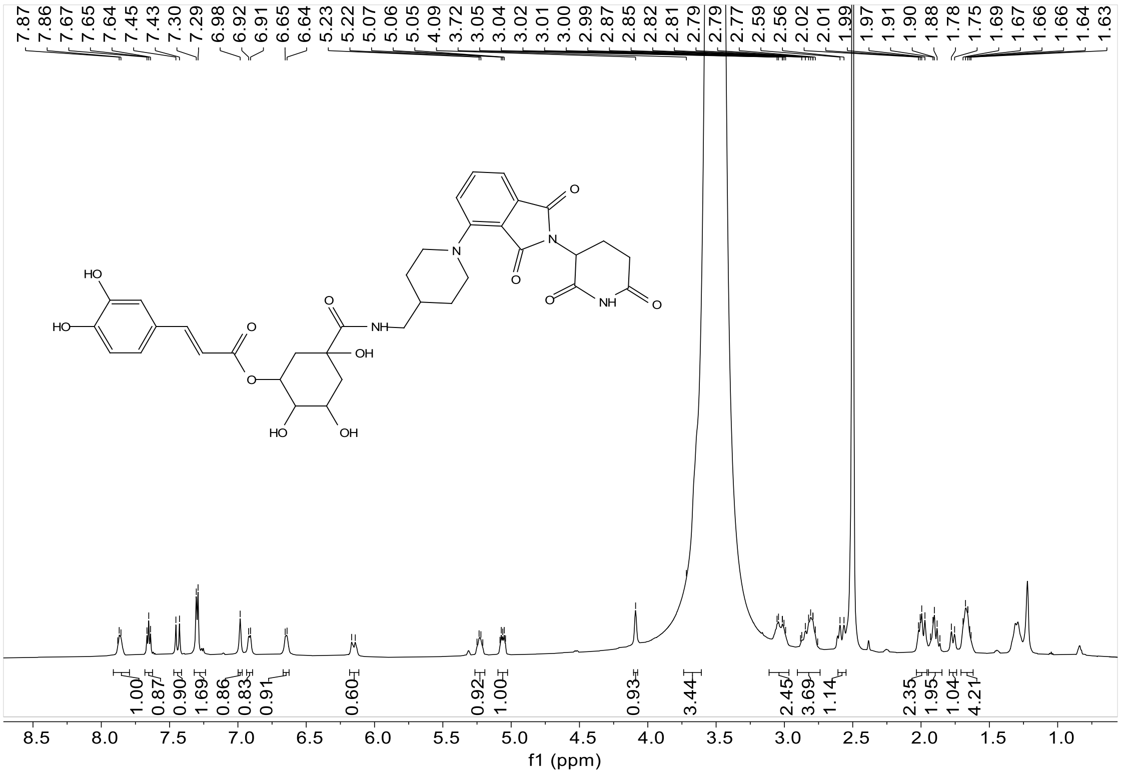

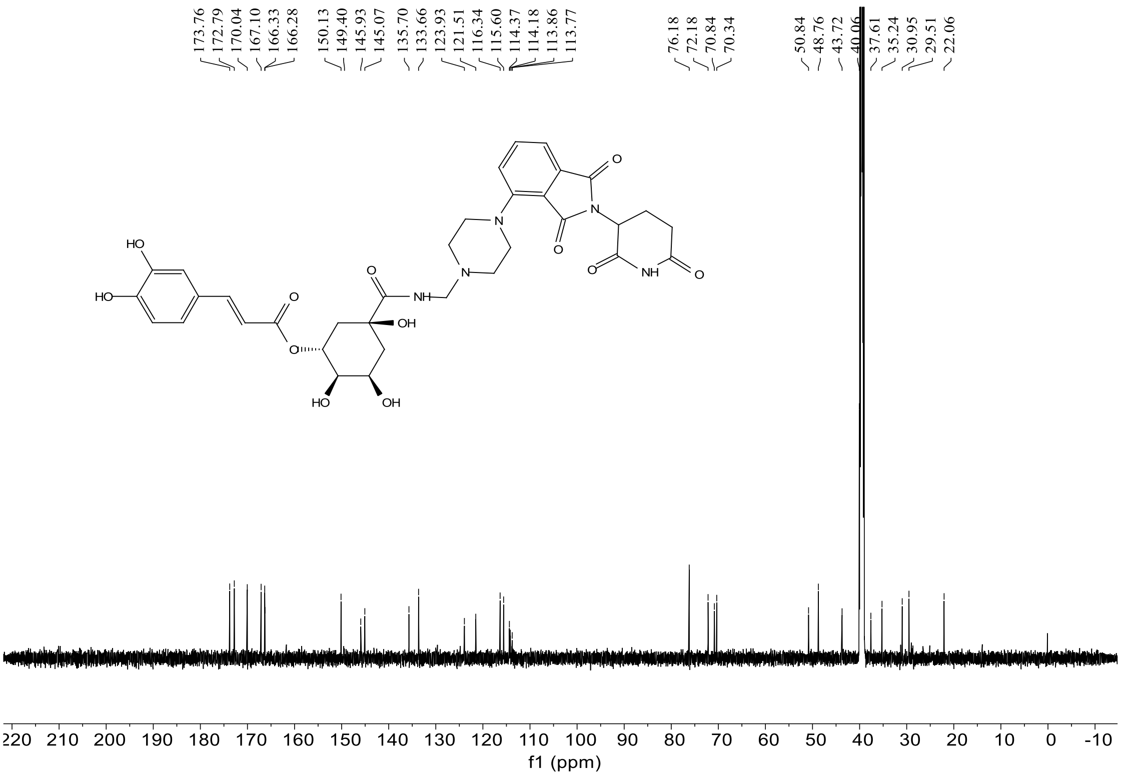

# Figure S2. Purity characterization data of compounds A1-A10

Compound A1 was detected for purity by liquid chromatography, using 0.1% formic acid water acetonitrile as the mobile phase system, chromatographic grade methanol as the solvent, and methanol as the solvent to dissolve an appropriate amount

of the product. Perform purity testing on it using Agilent 1290 ultra-high performance liquid chromatography. Retention time tracking was performed in the UV absorption band at 254nm, using Supersil ODS2 (2.1 mm × 100mm; 2 μ m) as the chromatographic column and a flow rate of 0.3mL/min. The purity of the sample was determined using the instrument's built-in integration function.

## 2.1 Purity characterization of Compound A1 (94.65%)

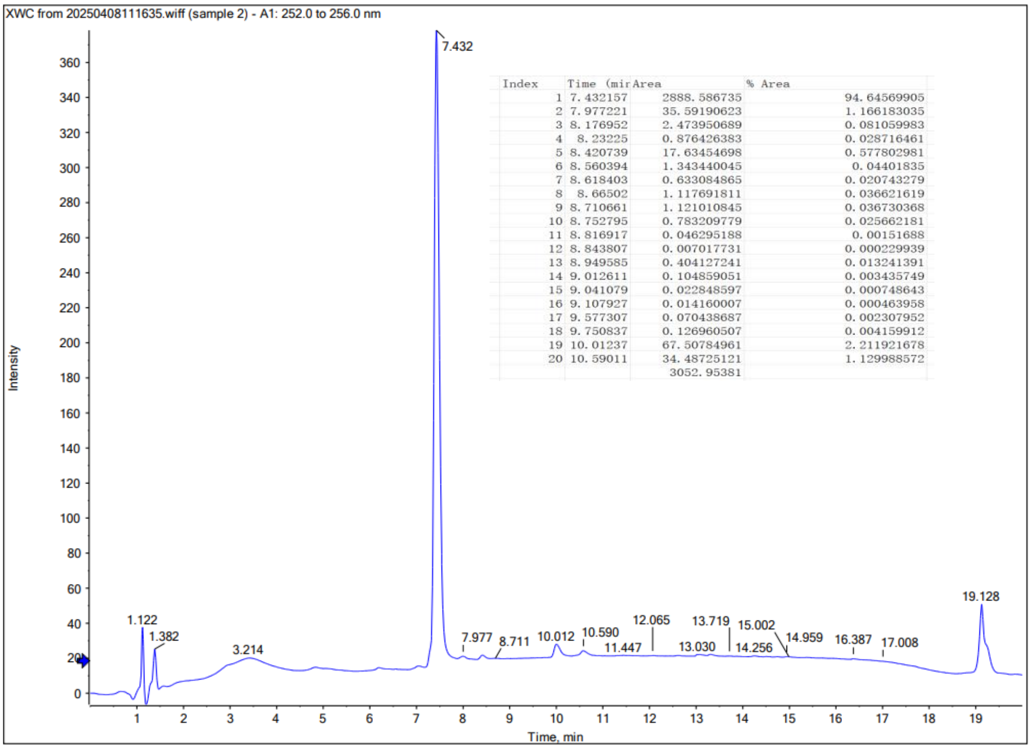


| Index | Time (mir | Area | % Area |
|---|---|---|---|
| 1 | 7.432157 | 2888.586735 | 94.64569905 |
| 2 | 7.977221 | 35.59190623 | 1.166183035 |
| 3 | 8.176952 | 2.473950689 | 0.081059983 |
| 4 | 8.23225 | 0.876426383 | 0.028716461 |
| 5 | 8.420739 | 17.63454698 | 0.577802981 |
| 6 | 8.560394 | 1.343440045 | 0.04401835 |
| 7 | 8.618403 | 0.633084865 | 0.020743279 |
| 8 | 8.66502 | 1.117691811 | 0.036621619 |
| 9 | 8.710661 | 1.121010845 | 0.036730368 |
| 10 | 8.752795 | 0.783209779 | 0.025662181 |
| 11 | 8.816917 | 0.046295188 | 0.00151688 |
| 12 | 8.843807 | 0.007017731 | 0.000229939 |
| 13 | 8.949585 | 0.404127241 | 0.013241391 |
| 14 | 9.012611 | 0.104859051 | 0.003435749 |
| 15 | 9.041079 | 0.022848597 | 0.000748643 |
| 16 | 9.107927 | 0.014160007 | 0.000463958 |
| 17 | 9.577307 | 0.070438687 | 0.002307952 |
| 18 | 9.750837 | 0.126960507 | 0.004159912 |
| 19 | 10.01237 | 67.50784961 | 2.211921678 |
| 20 | 10.59011 | 34.48725121 | 1.129988572 |
| | | 3052.95381 | |

## 2.2 Purity characterization of Compound A2 (95.34%)

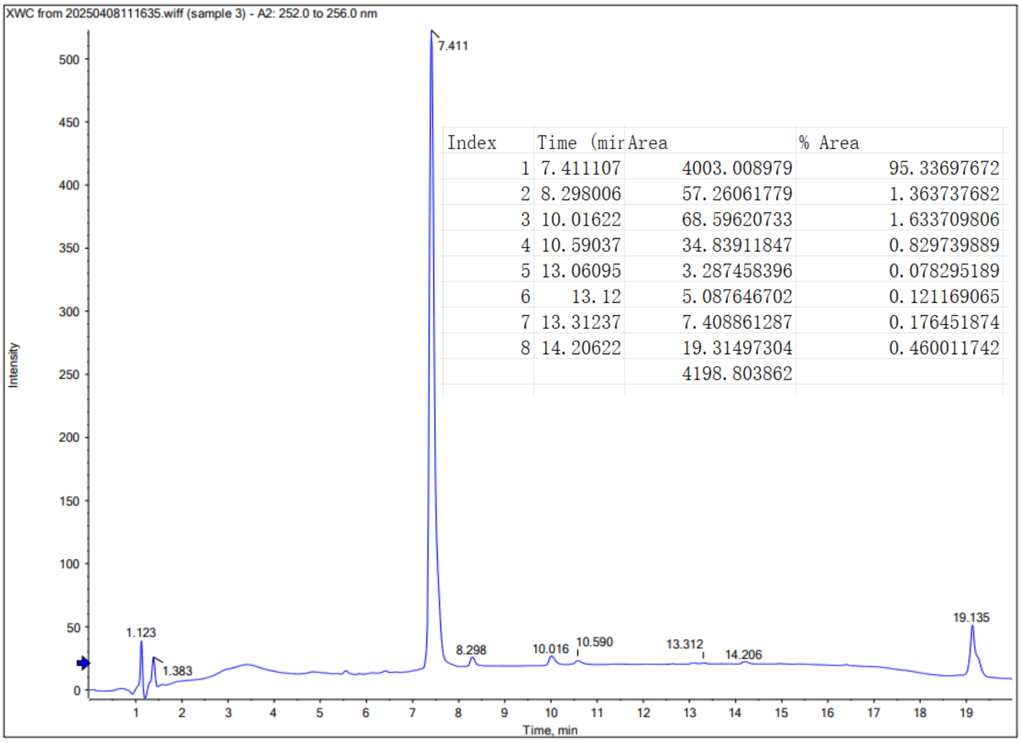


| Index | Time (mir | Area | % Area |
|---|---|---|---|
| 1 | 7.411107 | 4003.008979 | 95.33697672 |
| 2 | 8.298006 | 57.26061779 | 1.363737682 |
| 3 | 10.01622 | 68.59620733 | 1.633709806 |
| 4 | 10.59037 | 34.83911847 | 0.829739889 |
| 5 | 13.06095 | 3.287458396 | 0.078295189 |
| 6 | 13.12 | 5.087646702 | 0.121169065 |
| 7 | 13.31237 | 7.408861287 | 0.176451874 |
| 8 | 14.20622 | 19.31497304 | 0.460011742 |
| | | 4198.803862 | |

## 2.3 Purity characterization of Compound A3 (94.48%)

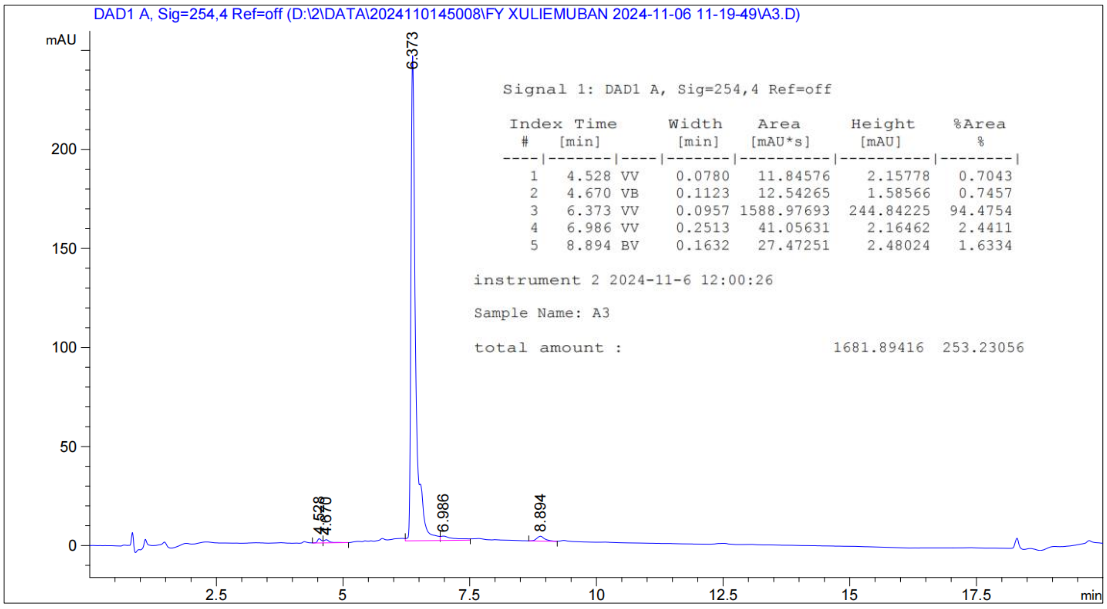


| Index # | Time [min] | | Width [min] | Area [mAU*s] | Height [mAU] | %Area % |
|---|---|---|---|---|---|---|
| 1 | 4.528 | VV | 0.0780 | 11.84576 | 2.15778 | 0.7043 |
| 2 | 4.670 | VB | 0.1123 | 12.54265 | 1.58566 | 0.7457 |
| 3 | 6.373 | VV | 0.0957 | 1588.97693 | 244.84225 | 94.4754 |
| 4 | 6.986 | VV | 0.2513 | 41.05631 | 2.16462 | 2.4411 |
| 5 | 8.894 | BV | 0.1632 | 27.47251 | 2.48024 | 1.6334 |
| total amount : | | | | 1681.89416 | 253.23056 | |

## 2.4 Purity characterization of Compound A4 (92.10%)

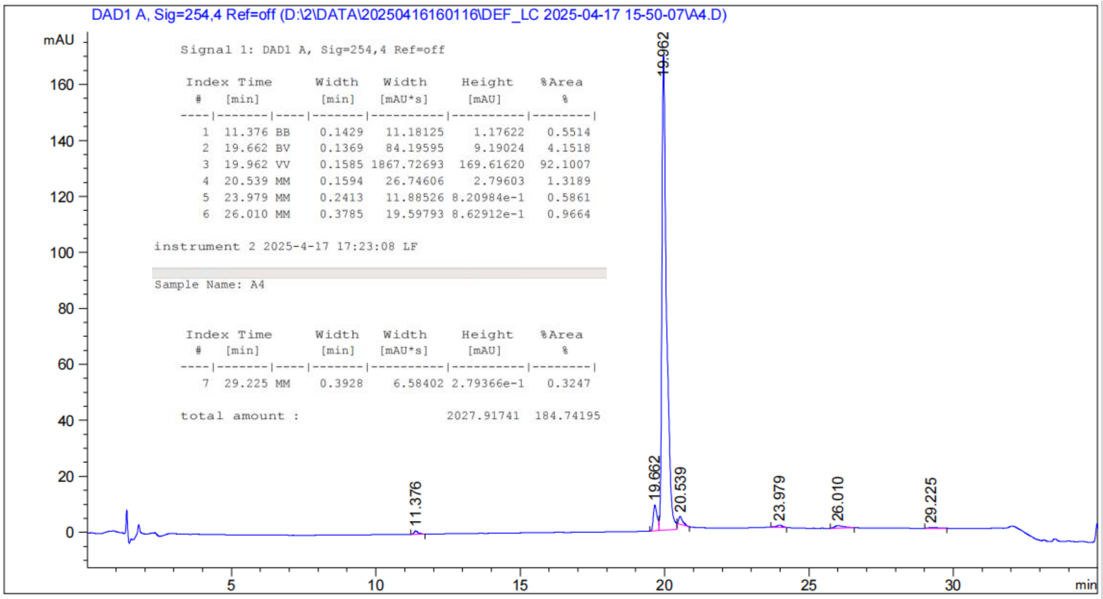


## 2.5 Purity characterization of Compound A5 (95.23%)

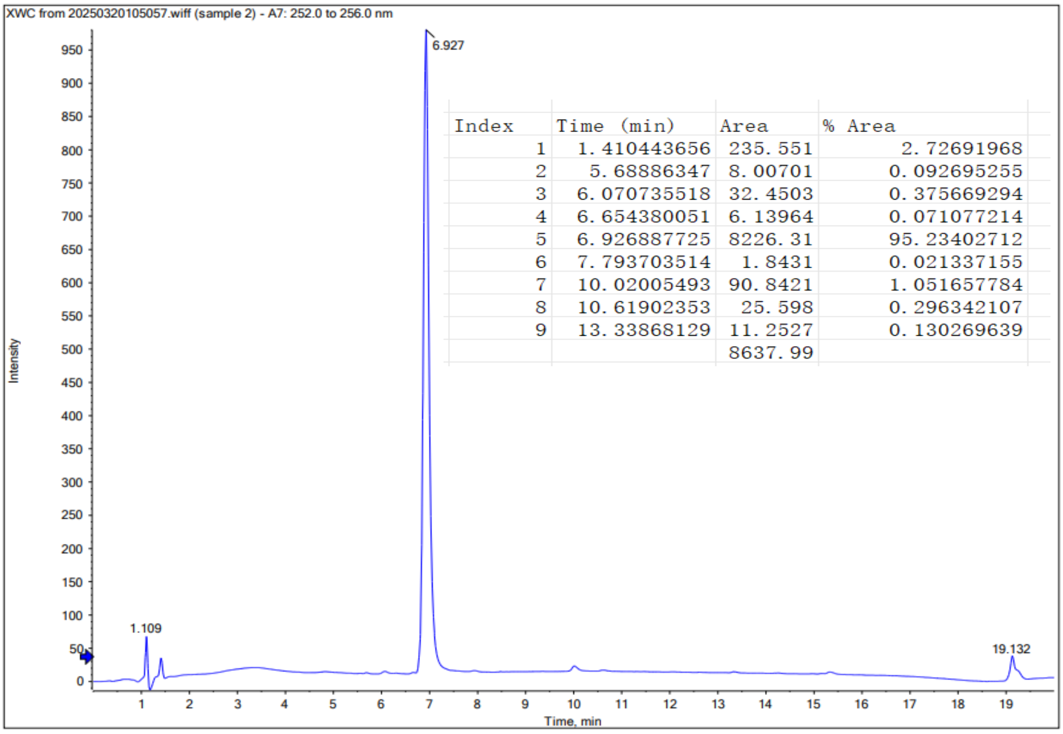


3/21/2025 11:56:47 AM

## 2.6 Purity characterization of Compound A6 (91.74%)

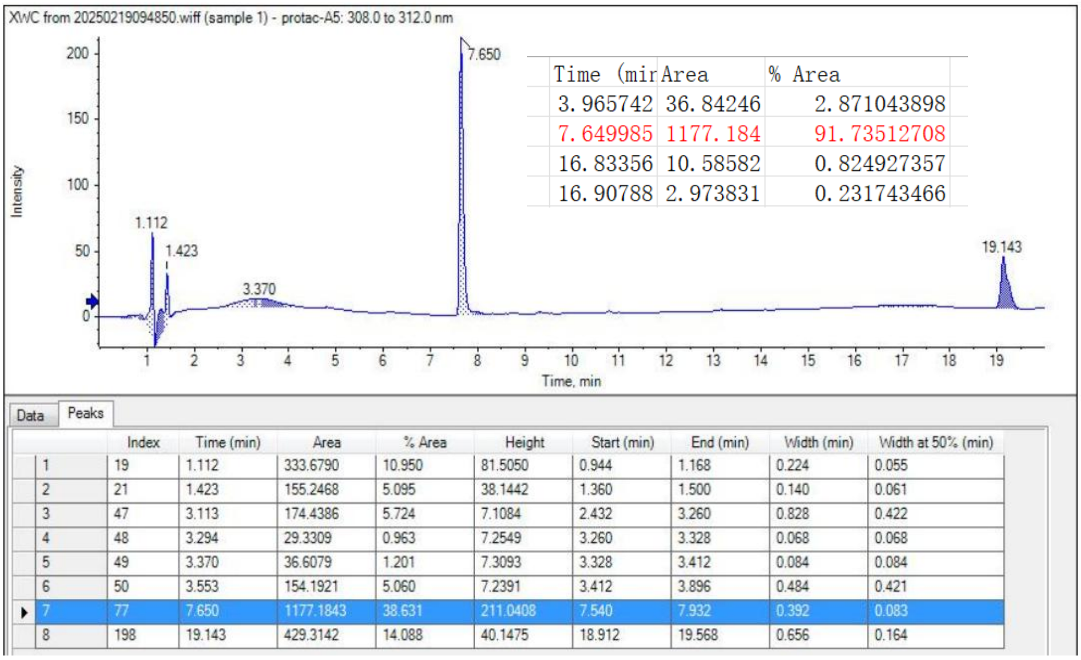


| Time (min | Area | % Area |
|---|---|---|
| 3.965742 | 36.84246 | 2.871043898 |
| 7.649985 | 1177.184 | 91.73512708 |
| 16.83356 | 10.58582 | 0.824927357 |
| 16.90788 | 2.973831 | 0.231743466 |

| | Index | Time (min) | Area | % Area | Height | Start (min) | End (min) | Width (min) | Width at 50% (min) |
|---|---|---|---|---|---|---|---|---|---|
| 1 | 19 | 1.112 | 333.6790 | 10.950 | 81.5050 | 0.944 | 1.168 | 0.224 | 0.055 |
| 2 | 21 | 1.423 | 155.2468 | 5.095 | 38.1442 | 1.360 | 1.500 | 0.140 | 0.061 |
| 3 | 47 | 3.113 | 174.4386 | 5.724 | 7.1084 | 2.432 | 3.260 | 0.828 | 0.422 |
| 4 | 48 | 3.294 | 29.3309 | 0.963 | 7.2549 | 3.260 | 3.328 | 0.068 | 0.068 |
| 5 | 49 | 3.370 | 36.6079 | 1.201 | 7.3093 | 3.328 | 3.412 | 0.084 | 0.084 |
| 6 | 50 | 3.553 | 154.1921 | 5.060 | 7.2391 | 3.412 | 3.896 | 0.484 | 0.421 |
| 7 | 77 | 7.650 | 1177.1843 | 38.631 | 211.0408 | 7.540 | 7.932 | 0.392 | 0.083 |
| 8 | 198 | 19.143 | 429.3142 | 14.088 | 40.1475 | 18.912 | 19.568 | 0.656 | 0.164 |

## 2.7 Purity characterization of Compound A7 (94.37%)

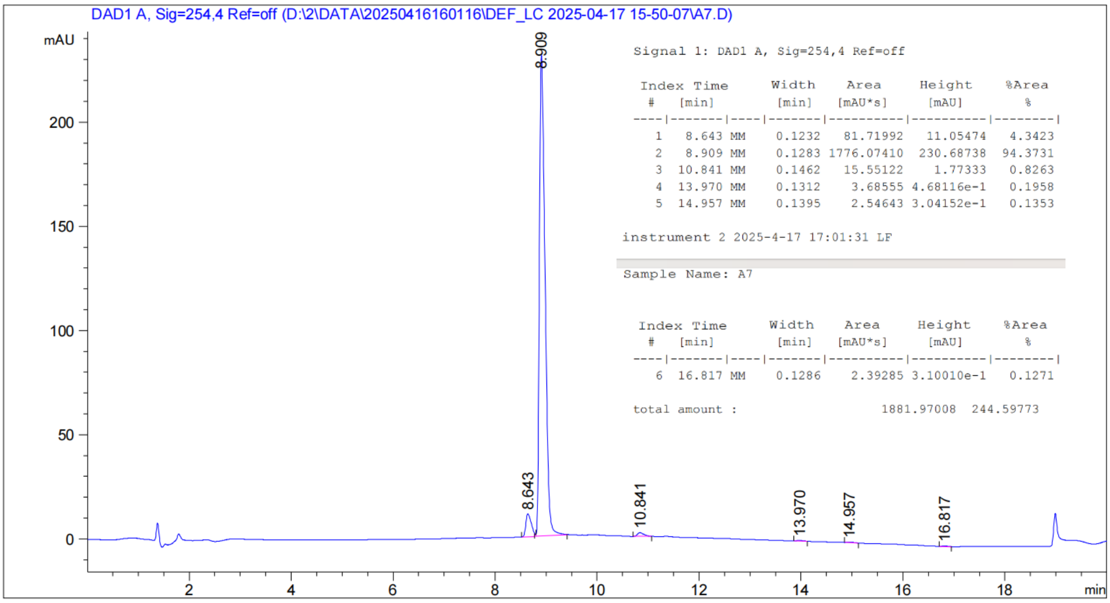


| Index # | Time [min] | | Width [min] | Area [mAU*s] | Height [mAU] | %Area % |
|---|---|---|---|---|---|---|
| 1 | 8.643 | MM | 0.1232 | 81.71992 | 11.05474 | 4.3423 |
| 2 | 8.909 | MM | 0.1283 | 1776.07410 | 230.68738 | 94.3731 |
| 3 | 10.841 | MM | 0.1462 | 15.55122 | 1.77333 | 0.8263 |
| 4 | 13.970 | MM | 0.1312 | 3.68555 | 4.68116e-1 | 0.1958 |
| 5 | 14.957 | MM | 0.1395 | 2.54643 | 3.04152e-1 | 0.1353 |

| Index # | Time [min] | | Width [min] | Area [mAU*s] | Height [mAU] | %Area % |
|---|---|---|---|---|---|---|
| 6 | 16.817 | MM | 0.1286 | 2.39285 | 3.10010e-1 | 0.1271 |

## 2.8 Purity characterization of Compound A8 (90.71%)

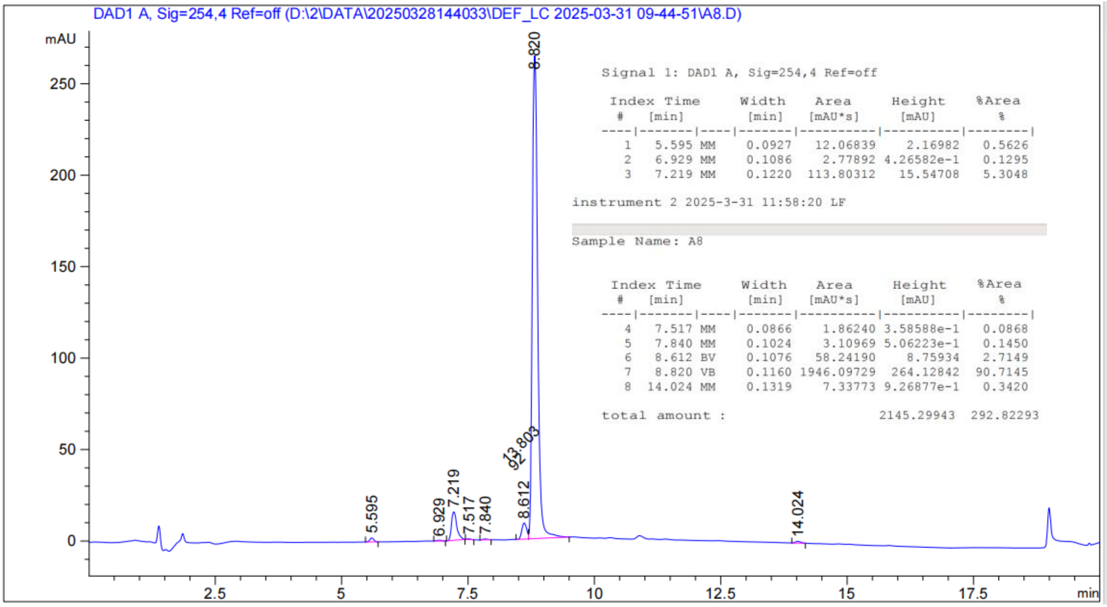


| Index # | Time [min] | | Width [min] | Area [mAU*s] | Height [mAU] | %Area % |
|---|---|---|---|---|---|---|
| 1 | 5.595 | MM | 0.0927 | 12.06839 | 2.16982 | 0.5626 |
| 2 | 6.929 | MM | 0.1086 | 2.77892 | 4.26582e-1 | 0.1295 |
| 3 | 7.219 | MM | 0.1220 | 113.80312 | 15.54708 | 5.3048 |
| 4 | 7.517 | MM | 0.0866 | 1.86240 | 3.58588e-1 | 0.0868 |
| 5 | 7.840 | MM | 0.1024 | 3.10969 | 5.06223e-1 | 0.1450 |
| 6 | 8.612 | BV | 0.1076 | 58.24190 | 8.75934 | 2.7149 |
| 7 | 8.820 | VB | 0.1160 | 1946.09729 | 264.12842 | 90.7145 |
| 8 | 14.024 | MM | 0.1319 | 7.33773 | 9.26877e-1 | 0.3420 |

## 2.9 Purity characterization of Compound A9 (91.44%)

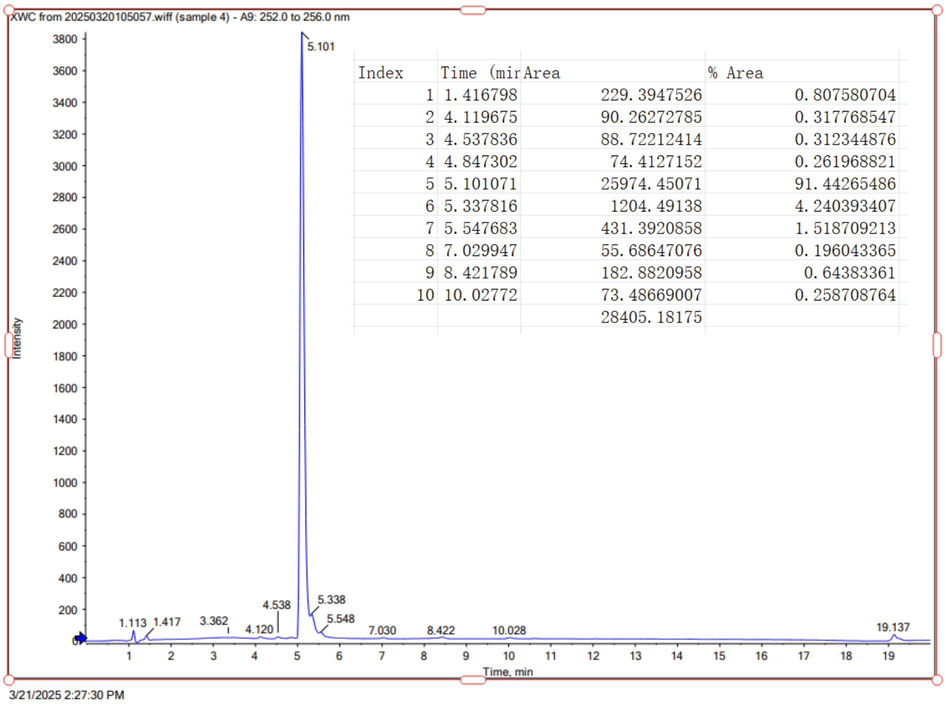


| Index | Time (min | Area | % Area |
|---|---|---|---|
| 1 | 1. 416798 | 229. 3947526 | 0. 807580704 |
| 2 | 4. 119675 | 90. 26272785 | 0. 317768547 |
| 3 | 4. 537836 | 88. 72212414 | 0. 312344876 |
| 4 | 4. 847302 | 74. 4127152 | 0. 261968821 |
| 5 | 5. 101071 | 25974. 45071 | 91. 44265486 |
| 6 | 5. 337816 | 1204. 49138 | 4. 240393407 |
| 7 | 5. 547683 | 431. 3920858 | 1. 518709213 |
| 8 | 7. 029947 | 55. 68647076 | 0. 196043365 |
| 9 | 8. 421789 | 182. 8820958 | 0. 64383361 |
| 10 | 10. 02772 | 73. 48669007 | 0. 258708764 |
| | | 28405. 18175 | |

## 2.10 Purity characterization of Compound A10 (93.87%)

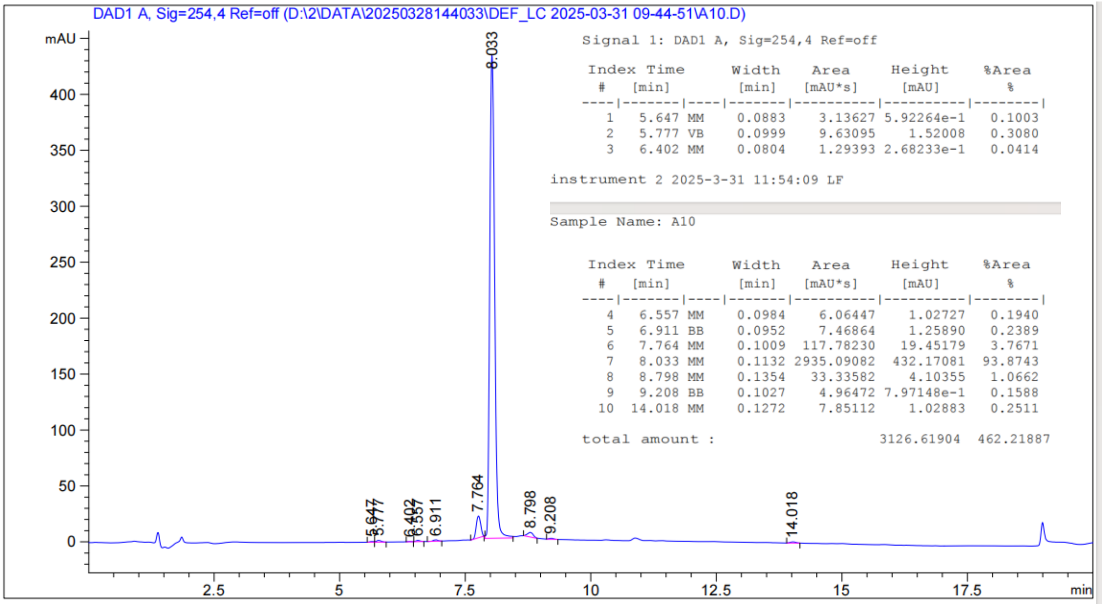


# Figure S3. HRMS spectra of all new compounds A1-A10

Using UHPLC30A-5600+liquid chromatography tandem quadrupole time-of-flight mass spectrometer to measure the mass spectrometry data of the target product at 35℃. Detecting mass spectrometry data in positive ion mode.

## 3.1 HRMS spectra of Compound A1 (696)

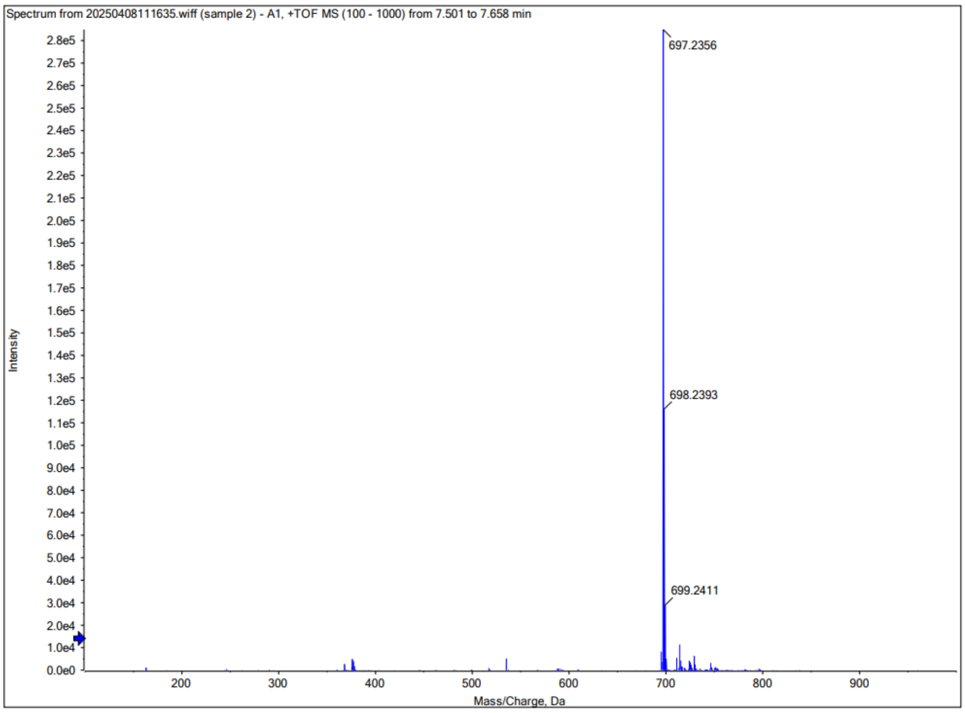


## 3.2 HRMS spectra of Compound A2 (740)

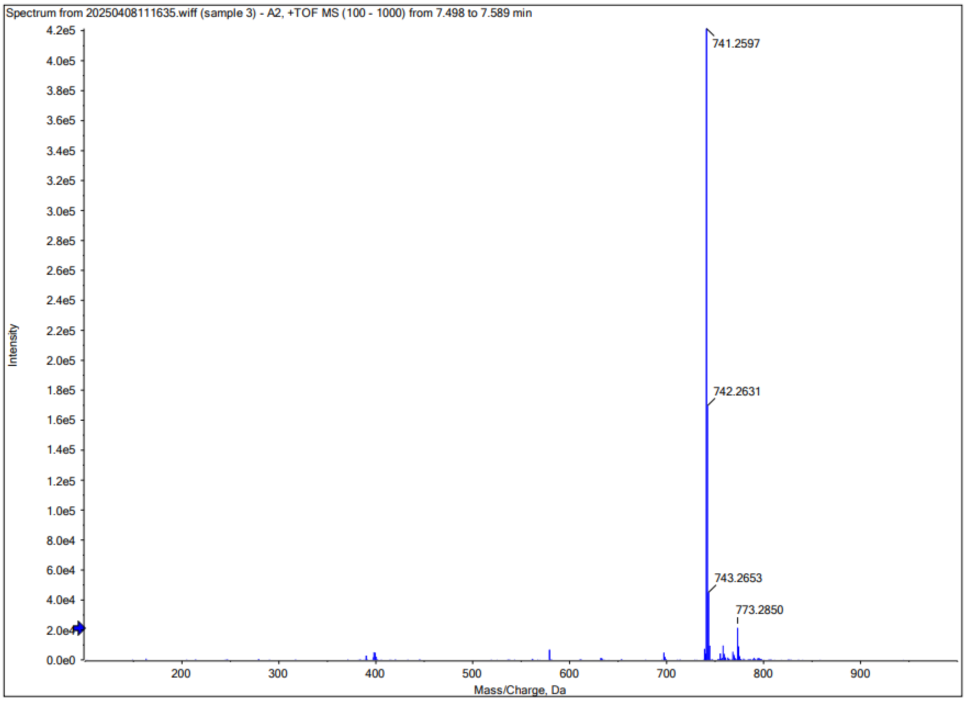


## 3.3 HRMS spectra of Compound A3 (784)

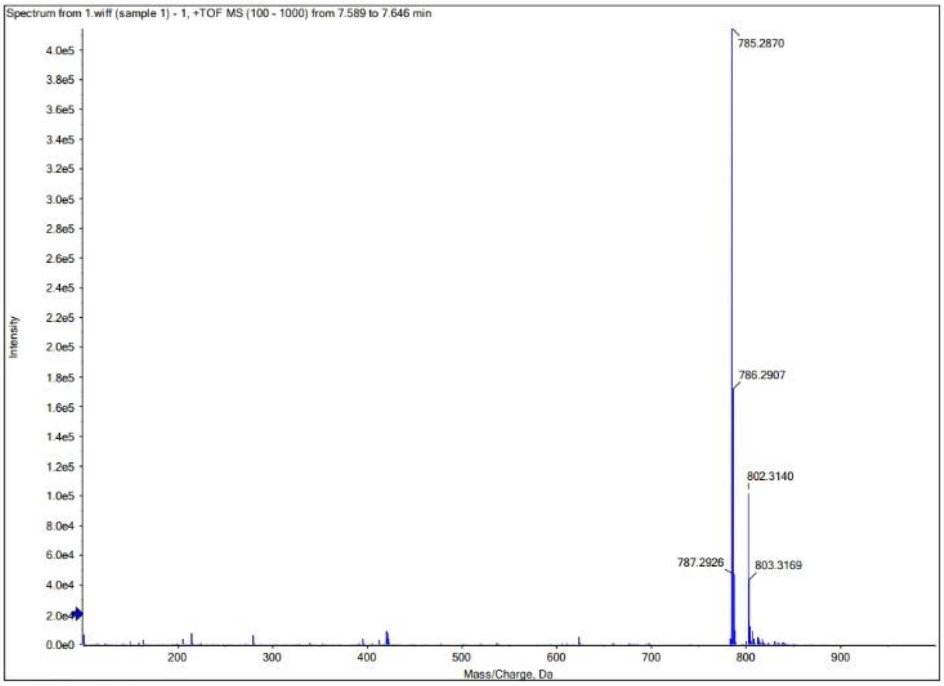


9/29/2024 3:00:08 PM

## 3.4 HRMS spectra of Compound A4 (828)

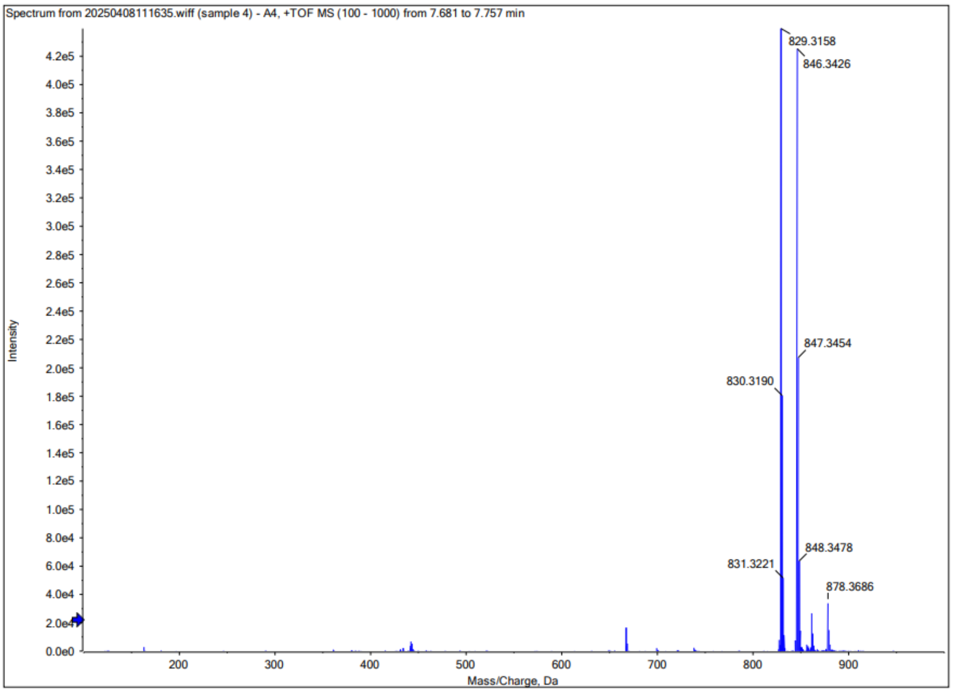


## 3.5 HRMS spectra of Compound A5 (652)

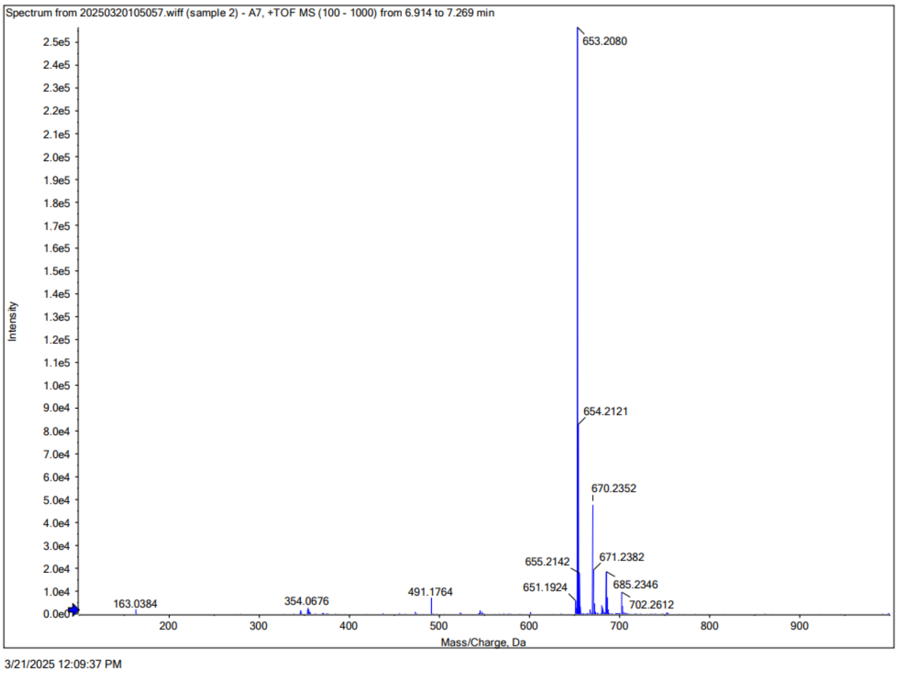


## 3.6 HRMS spectra of Compound A6 (680)

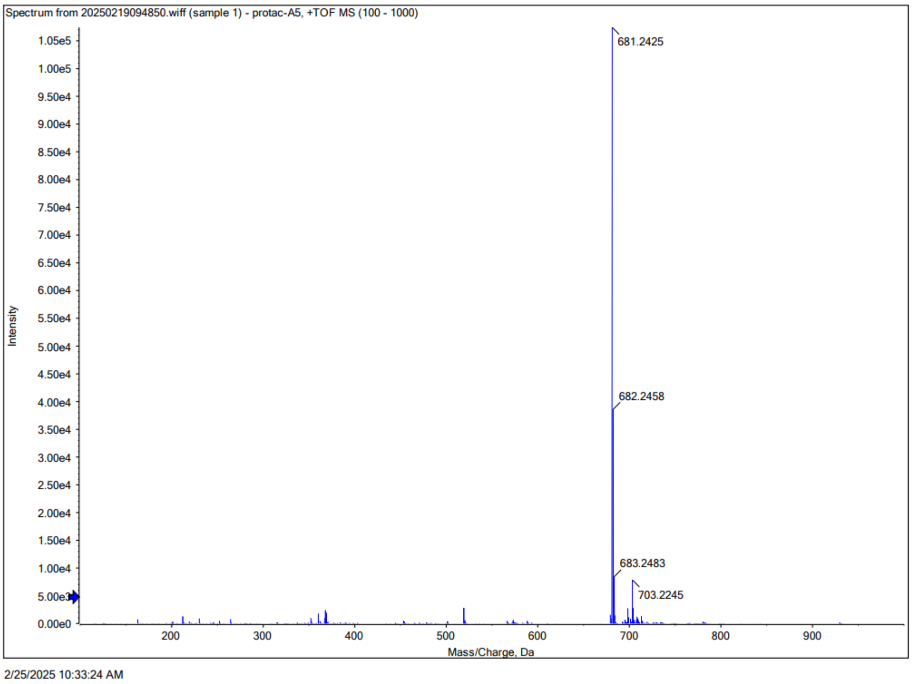


## 3.7 HRMS spectra of Compound A7 (708)

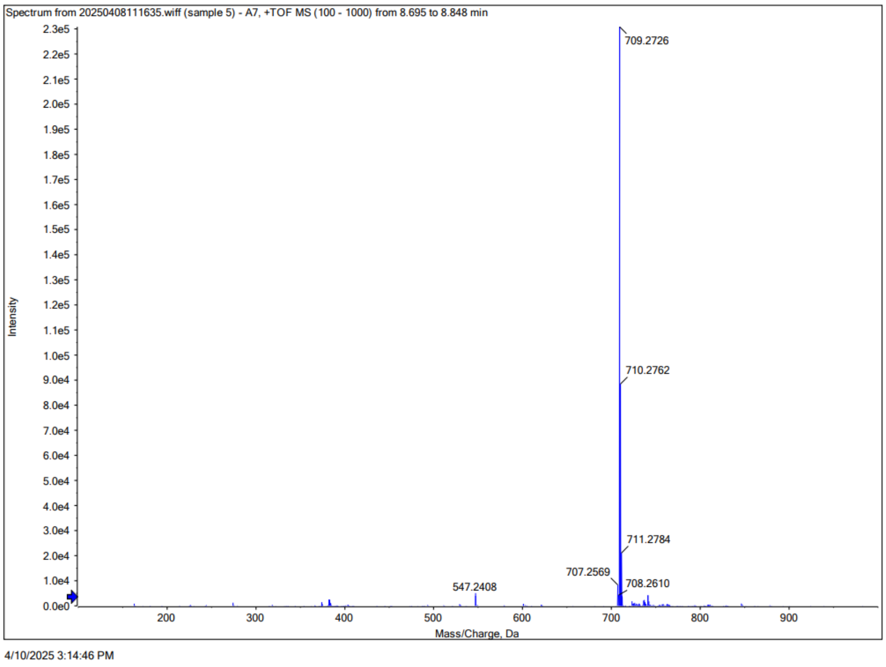


## 3.8 HRMS spectra of Compound A8 (728)

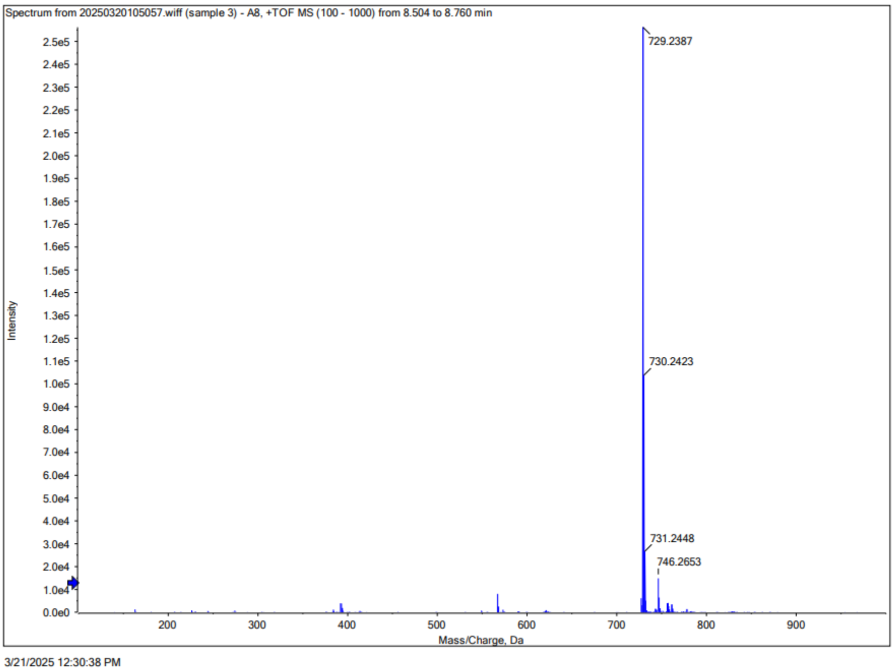


## 3.9 HRMS spectra of Compound A9 (721)

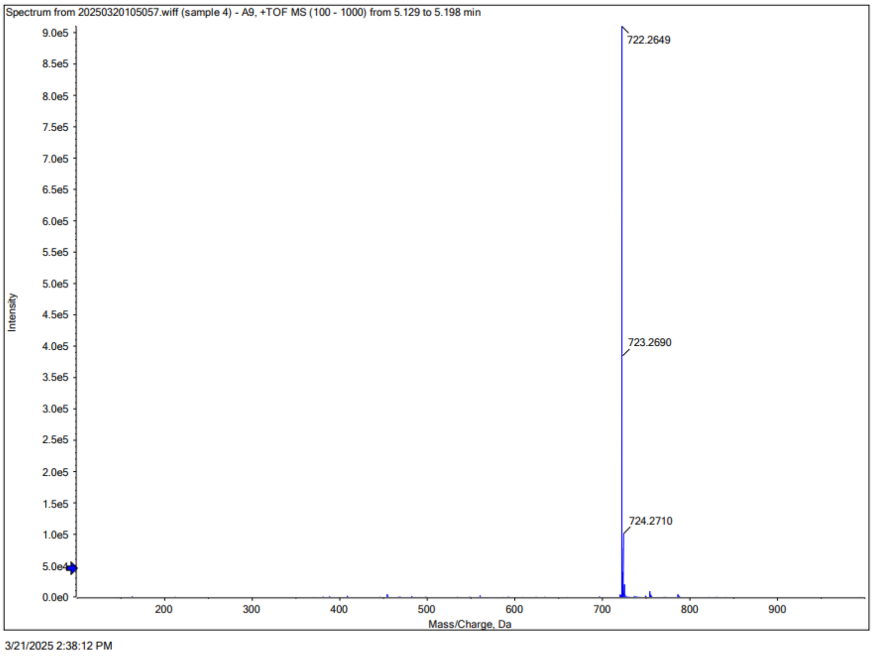


## 3.10 HRMS spectra of Compound A10 (706)

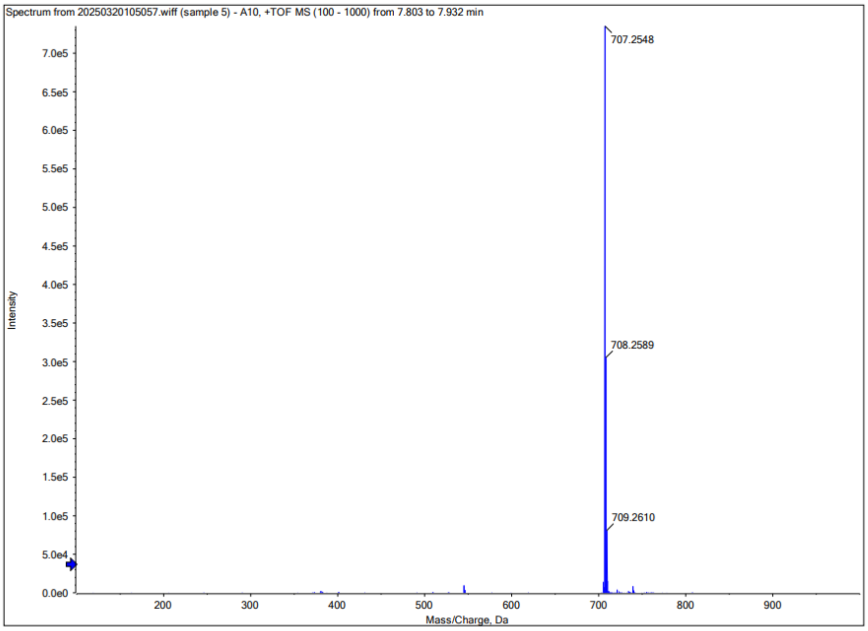


# Table S1. Proteins significantly downregulated in proteomics

| GeneName | P-VALUE | P-VALUE-chitest | P-VALUE(plot) | LOG_FOLDCHANGE |
|---|---|---|---|---|
| FIZ1 | 0.00302566 | 0.014305878 | 0.00302566 | -16.96777233 |
| DIPK1B | 0.006333411 | 0.014305878 | 0.006333411 | -14.50494102 |
| RAB28 | 0.001164333 | 0.014305878 | 0.001164333 | -14.06512396 |
| CDC42EP5 | 0.000704351 | 0.014305878 | 0.000704351 | -13.22254411 |
| ZNF584 | 0.022472448 | 0.014305878 | 0.022472448 | -12.84726556 |
| EN2 | 0.005076984 | 0.014305878 | 0.005076984 | -12.42500517 |
| CELSR2 | 0.017113934 | 0.014305878 | 0.017113934 | -11.5098714 |
| UPP2 | 0.074901541 | 0.014305878 | 0.014305878 | -11.0166578 |
| ZP4 | 0.015090946 | 0.014305878 | 0.015090946 | -10.61563947 |
| ZNF653 | 0.043503482 | 0.014305878 | 0.043503482 | -10.17030472 |
| CCSER2 | 0.053648143 | 0.014305878 | 0.014305878 | -10.09675577 |
| MCTS2 | 0.005233247 | 0.014305878 | 0.005233247 | -8.05616616 |
| MDM4 | 0.020708577 | 0.014305878 | 0.020708577 | -6.810047092 |
| TCEAL8 | 0.00854308 | 0.014305878 | 0.00854308 | -5.037565429 |
| PDZD2 | 0.010853783 | 0.014305878 | 0.010853783 | -4.875912467 |
| ZBED4 | 0.00151898 | 0.083264517 | 0.00151898 | -3.347438588 |
| ZNF827 | 0.002304696 | 0.083264517 | 0.002304696 | -3.332005952 |
